# Metabolomic and Lipidomic Profiling Identifies The Role of the RNA Editing Pathway in Endometrial Carcinogenesis

**DOI:** 10.1038/s41598-017-09169-2

**Published:** 2017-08-18

**Authors:** Tatiana Altadill, Tyrone M. Dowdy, Kirandeep Gill, Armando Reques, Smrithi S. Menon, Cristian P. Moiola, Carlos Lopez-Gil, Eva Coll, Xavier Matias-Guiu, Silvia Cabrera, Angel Garcia, Jaume Reventos, Stephen W. Byers, Antonio Gil-Moreno, Amrita K. Cheema, Eva Colas

**Affiliations:** 1 Biomedical Research Group in Gynecology, Vall Hebron Research Institute (VHIR), Universitat Autònoma de Barcelona, CIBERONC Barcelona, Spain; 2 Department of Oncology, Georgetown University Medical Center, Washington D.C., USA; 3 Pathology Department, Vall Hebron University Hospital, Barcelona, Spain; 4 Pathological Oncology Group and Pathology Department, University Hospital Arnau de Vilanova, and University Hospital Bellvitge, IRBLLEIDA and Idibell, University of Lleida, CIBERONC Lleida, Spain; 5 Gynecological Oncology Department, Vall Hebron University Hospital, Barcelona, Spain; 6 Basic Sciences Department, International University of Catalonia, CIBERONC Barcelona, Spain; 7 Department of Biochemistry Molecular and Cellular Biology, Georgetown-Lombardi Comprehensive Cancer Center, Washington, D.C., United States

## Abstract

Endometrial cancer (EC) remains the most common malignancy of the genital tract among women in developed countries. Although much research has been performed at genomic, transcriptomic and proteomic level, there is still a significant gap in the metabolomic studies of EC. In order to gain insights into altered metabolic pathways in the onset and progression of EC carcinogenesis, we used high resolution mass spectrometry to characterize the metabolomic and lipidomic profile of 39 human EC and 17 healthy endometrial tissue samples. Several pathways including lipids, Kynurenine pathway, endocannabinoids signaling pathway and the RNA editing pathway were found to be dysregulated in EC. The dysregulation of the RNA editing pathway was further investigated in an independent set of 183 human EC tissues and matched controls, using orthogonal approaches. We found that ADAR2 is overexpressed in EC and that the increase in expression positively correlates with the aggressiveness of the tumor. Furthermore, silencing of ADAR2 in three EC cell lines resulted in a decreased proliferation rate, increased apoptosis, and reduced migration capabilities *in vitro*. Taken together, our results suggest that ADAR2 functions as an oncogene in endometrial carcinogenesis and could be a potential target for improving EC treatment strategies.

## Introduction

Endometrial cancer (EC) accounts for 7% of the new cases of female cancers in United States in 2017 and its incidence is increasing^1, 2^. The most standardized classification divides EC in two different subtypes: type I or endometrioid carcinomas (EEC), which is the most frequent subtype; and type II or non-endometrioid carcinomas (NEEC), which are more aggressive tumors. Among NEEC subtypes, serous is the most prominent histology^3, 4^. Moreover, EC tumors are classified according to the extent of tumor dissemination (International Federation of Gynecology and Obstetrics or FIGO staging) and histological grade^1^. About 20% of patients are diagnosed at an advanced stage and/or at a high histological tumor grade and have a low 5-year survival rate associated^5, 6^. As such, availability of biomarkers for disease stratification and a thorough understanding of biochemical perturbations that underscore EC progression are likely to improve clinical outcomes.

Extensive research performed using high-throughput technologies including genomics, transcriptomics and proteomics has augmented characterization of molecular changes at different levels of cellular expression that are specifically associated with human endometrium^7^ and onset and progression of EC^8, 9^. Metabolomics defines the end point of cellular processes and hence provides a readout of current physiological status of the system^10^. Thus, metabolomics, lipidomics and glycomics have emerged as promising tools for clinical and translational research^11^. Rapid advancement of metabolomics technologies such as ultra-performance liquid chromatography mass spectrometry (UPLC-MS), enable comprehensive interrogation of the human metabolome and lipidome^12–15^. Bioinformatics analyses of these data is likely to augment the discovery of new clinical and pharmacological targets^16^.

It is known that changes at the transcriptomic levels generate a new source of complexity that promotes initiation and progression of cancer and other diseases^17–19^. The most common RNA editing events are mediated post-transcriptionally by the Adenosine deaminases acting on RNA (ADAR) family of enzymes^20^. The ADAR gene family catalyzes the deamination of adenosine that is converted to an inosine creating a dysregulation of the adenosine/inosine (A/I) ratio in the cell. Adenosine to inosine (A-to-I) editing can lead to amino acid recoding events^21^ since inosine is recognized as a guanosine. This family includes three enzymes, ADAR1 (UniProtKB P55265), ADAR2 (UniProtKB P78563) and ADAR3 (UniProtKB Q9NS39); also known as ADAR, ADARB1 and ADARB2, respectively^22^. ADAR1 and ADAR2 are ubiquitously expressed and, differently that the brain specific ADAR3, they show catalytic activity^23^.

The overall goal of this study was to identify altered metabolic pathways in EC. Characterization of the metabolome and lipidome of EC tumors was performed using a high resolution mass spectrometry approach in conjunction with UPLC-MS. Pathway validation was performed with an independent set of samples that for the first time, yielded insights into dysregulation of the RNA editing pathway in endometrial tumors. Alterations of the RNA editing pathway correlated with high histological grade and EC serous subtypes that are indicators of poor prognosis in EC. Finally, the expression of ADAR editing enzymes was modulated *in vitro* to confirm an important oncogenic role of this pathway in EC cell proliferation, apoptosis and migration. These results are instructive of the role of this pathway in EC tumor progression.

## Results

### Untargeted metabolomics profiling of EC human tissue samples

To better understand alterations in metabolic pathways occurring in EC, we performed metabolomics/lipidomics untargeted discovery using UPLC-ESI-TOF-MS of tumor and non-tumoral tissue samples. The sample set included a total of 56 samples: 39 EEC tumors from different FIGO stages, including 10 stages IA, 9 stages IB, 10 stages II and 10 stages III; and 17 benign endometrial tissues (see patient details in Table 1). All patients included in the study were postmenopausal women and did not receive any treatment before surgery. Pre-processing of TOFMS data yielded a total of 8,146 features in the positive and 7,558 in the negative electrospray ionization mode, respectively.

**Table 1:** Clinical and histopathological information of the patients included in this study.

**Diagnosis**	**Number of samples**	**FIGO stage**	**Type I/II**	**Pre/Postmenopausal**	**Phase**
Control tissue	17			Post	Discovery and verification
EC tissue	10	IA	I	Post	Discovery and verification
	9	IB	I	Post	Discovery and verification
	10	II	I	Post	Discovery and verification
	10	III	I	Post	Discovery and verification

**Figure 1:**
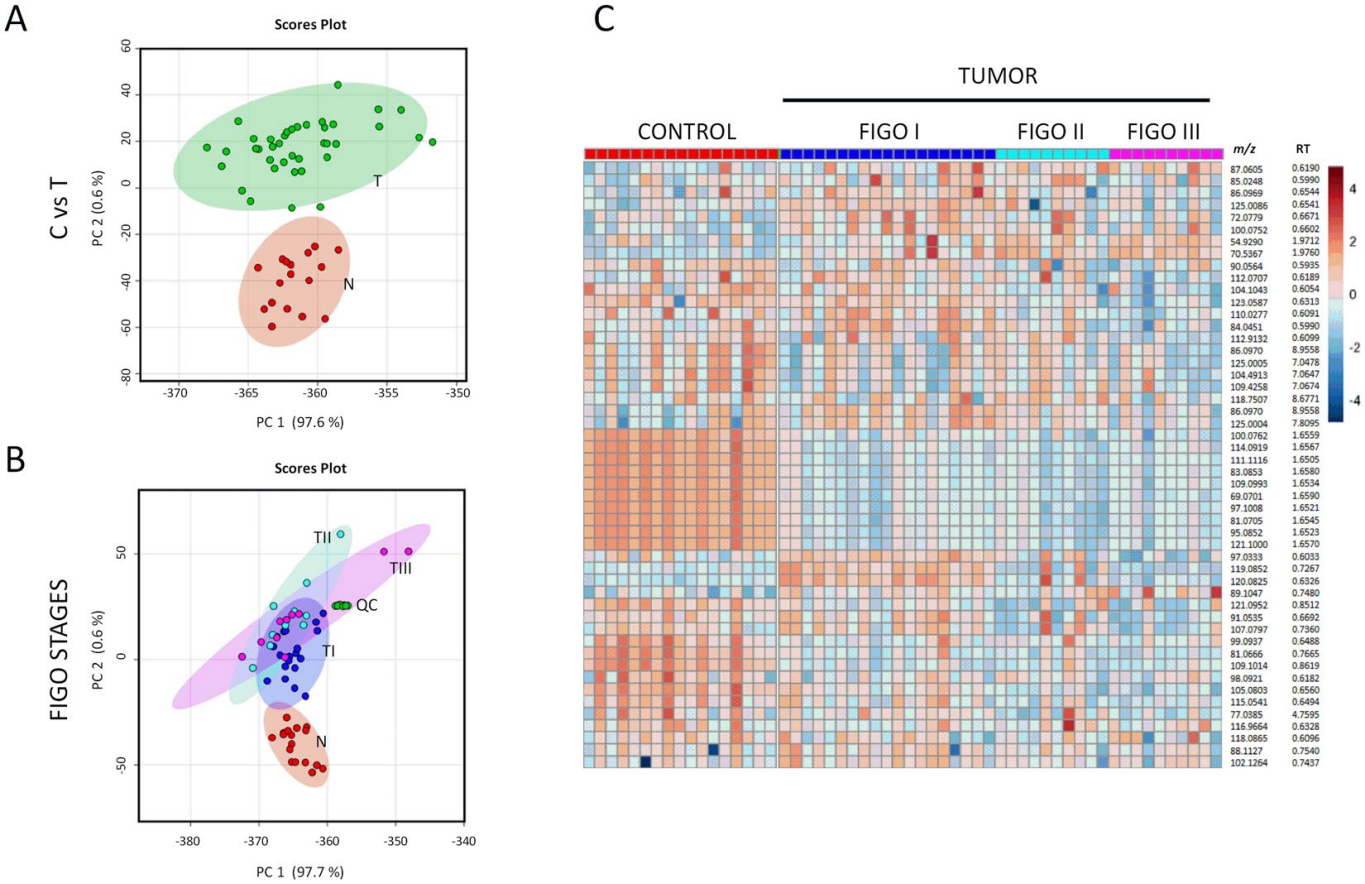
Multivariate analysis showing metabolic profiles in EC. Principal Component Analysis (PCA) plots showing separation between EC tissue samples (T, in green) and control tissue samples (N, in red) (Panel A) and separation between different EC FIGO stages (stage 1: TI, stage 2: TII, stage 3: TIII) (Panel B) for the positive MS ionization mode. X-axis shows interclass separation and Y-axis illustrates the intra-class variability. Panel C. Heat map of ion rankings, corresponding to their relative concentrations (intensity) for positive MS ionization mode, in the same cohort of subjects. Each row represents a unique feature with a specific *m/z* and RT while each column represents a unique subject. (*m/z*: mass to charge ratio; RT: retention time).

In order to define a generic metabolomic profile of EC, initially, we combined all tumor samples from EEC patients in one group (n = 39) and compared them against matched control tissues (n = 17). Inherent differences in metabolomic profiles were visualized using descriptive Principal Component Analysis (PCA) plot that showed clear separation between tumors and controls (Fig. 1A). Subsequently, t-statistics was used to select 80 metabolites that showed significant variation (adjusted p-value < 0.05) between the two study groups and fold change (FC) values over 2 and below 0.5 (Supplementary Table 1). We confirmed the putative identity of a subset of 42 metabolites using tandem mass spectrometry (MS/MS) (Table 2). An example of the fragmentation pattern of two verified metabolites is shown in Supplementary Figures 1 and 2. We found a significant dysregulation in the lipid metabolism, with an important number of glycerophosphocholines (PCs), phosphatidylserine (PSs), phosphatidylethanolamines (PEs), phosphatidylinositols (PIs), and phosphatidylglycerol (PGs) that were upregulated in the endometrial tumor tissue (detailed in Table 2) including PC (14:0/18:2), PC (16:0/20:4), PC (16:0/20:5), PC (16:0/22:6), PC (18:0/20:2), PC (18:1/14:0), and PC (18:1/22:6). Moreover, a total of 9 PEs (PE (16:0/22:6), PE (16:1/P-18:1), PE (18:0/0:0), PE (18:0/18:3), PE (18:1/22:6), PE (18:1/16:0), PE (18:1/18:1), PE (18:4/P-18:1) and PE (P-16:0/0:0)) and 4 PIs (PI (14:0/22:1), PI (16:0/18:1), PI (16:0/22:3) and PI (18:1/18:1)) showed significant changes in the relative abundance in EC tumors as compared to the matched controls. Metabolites such as linoleic acid, 3-Deoxyvitamin D3, UDP-N-acetyl-D-galactosamine and 1-Palmitoyl-2-linoleoyl PE were observed to be also upregulated whereas peptide Glu Phe Arg Trp, some amides (palmic amide, stearamide and oleamide), PA (18:0/18:1), PE (20:1/22:6), PE (22:6/P-18:1), PG (19:0/22:4) and other important metabolites such inosine and picolinic acid showed lower abundance in the tumor tissue.

**Table 2:** Potential biomarkers confirmed by MS/MS. The table lists metabolites that showed significant change in the relative abundance in EC tissues compared to controls. (*m/z* = mass/charge; ppm = parts per million; RT = retention time; p-v = p-value; FC = fold change; FDR = false discovery rate; AUC = area under the curve).

**Metabolite**	***m/z***	**Mass error (ppm)**	**RT (min)**	**In EC compared to Control**	**FC (T/C)**	**p-v**	**FDR**	**AUC**	**Formula**	**Ionization mode**	**Major CID fragments**
Picolinic acid	122.02446	2	0.6499	↓	0.1834	3.07E-03	3.47E-03	0.8275	C6H5NO2	Negative	107.01, 105.02, 87.12, 81.53
Linoleic acid	279.2320	3	6.9054	↑	3.2817	2.33E-02	2.33E-02	0.6772	C18H32O2	Negative	240.99, 170.99, 96.96
Vaccenic acid	281.2482	1	7.3972	↓	0.12817	1.65E-07	4.73E-07	0.6352	C18H34O2	Negative	94.99, 96.96, 100.99, 126.90
Arachidonic Acid (peroxide free)	303.2325	1	6.8246	↓	0.29314	9,94E-04	1.30E-03	0.6439	C20H32O2	Negative	259.25, 216.99, 205.19
5,8,11-eicosatrienoic acid	305.2481	1	7.1599	↑	19.139	2.28E-05	4.09E-05	0.6189	C20H34O2	Negative	216.99, 287.00, 216.98, 1165.93, 78.98
PE(P-16:0/0:0)	436.2827	1	5.8143	↑	8.4545	1.82E-03	2.24E-03	0.7984	C21H44NO6P	Negative	78.96, 140.01, 196.03
PE(18:0/0:0)	480.3092	0	6.3049	↑	11.4850	7.95E-04	1.07E-03	0.8246	C23H48NO7P	Negative	96.96, 140.01, 196.04, 283.27
PG(22:6/0:0)	555.2716	2	5.6562	↑	5.2860	4.68E-03	5.16E-03	0.8024	C28H45O9P	Negative	170.98, 283.24, 327.23
UDP-N-acetyl-D-galactosamine	606.07389	0	0.5454	↑	59.8910	4.41E-06	9.15E-06	0.8695	C17H27N3O17 P2	Negative	111.0222, 158.92, 176.93, 282.04, 362.00, 385.00
PE(16:1/P-18:1)	698.5116	2	9.5703	↑	2.9631	9.80E-03	1.05E-02	0.7232	C39H74NO7P	Negative	78.96, 140.00, 253.21, 281.23
PA (18:0/18:1)	701.5236	0	9.8145	↓	0.1377	3.47E-06	7.85E-06	0.9056	C38H75N2O7P	Negative	281.25, 283.25, 419.25, 437.28
1-Palmitoyl-2-linoleoyl PE	714.5078	0	9.4018	↑	14.4588	1.16E-04	1.92E-04	0.8549	C39H74NO8P	Negative	140.01, 255.23, 279.22
PE(18:1/16:0)	716.5231	0	9.2768	↑	20.1049	6.31E-04	8.75E-04	0.8491	C39H76NO8P	Negative	140.01, 196.04, 281.25
PE(18:4/P-18:1)	720.4964	1	9.8168	↑	12.3568	1.50E-04	2.39E-04	0.859	C41H72NO7P	Negative	259.20, 581.49
PE(18:0/18:3)	740.5213	3	9.4586	↑	2.4862	8.10E-07	2.18E-06	0.8823	C41H76NO8P	Negative	140.01, 283.24, 482.32
PE(18:1/18:1)	742.5388	0	9.4276	↑	86.8130	2.11E-06	5.04E-06	0.9324	C41H78NO8P	Negative	140.01, 281.24, 460.28, 478.30
PG(O-16:0/20:1)	761.5677	3	9.1881	↑	2.4041	3.40E-04	5.04E-04	0.8392	C42H83O9P	Negative	152.99, 255.23, 391.20, 465.25
PE(16:0/22:6)	762.5106	3	9.9369	↑	2.5258	2.95E-04	4.53E-04	0.8322	C43H74NO8P	Negative	140.01, 255.23, 283.24, 327.23, 452.27
PE(22:6/P-18:1)	772.5278	1	9.3001	↓	0.2313	2.17E-03	2.59E-03	0.7861	C45H76NO7P	Negative	140.01, 283.24, 327.23, 444.28, 462.29
PS(13:0/22:1)	774.5303	1	8.9030	↑	76.5521	2.09E-09	1.12E-08	0.9307	C41H78NO10P	Negative	152.99, 491.31, 687.49
PE(18:1/22:6)	788.5258	2	9.1145	↑	8.9976	9.22E-05	1.59E-04	0.8939	C45H76NO8P	Negative	78.95 140.01, 152.99, 281.24, 283.26, 327.23, 460.26, 478.29, 506.26
PE(20:1/22:6)	816.5526	2	8.9397	↓	0.5173	2.94E-12	2.53E-11	0.979	C47H80NO8P	Negative	78.95, 152.99, 283.25, 309.27, 506.32
PI(16:0/18:1)	835.5348	0	9.7641	↑	20.4232	5.08E-06	9.93E-06	0.8881	C43H81O13P	Negative	78.95, 152.99, 281.25, 391.22, 553.29, 579.29
PG(19:0/22:4)	839.5768	4	9.8027	↓	0.4242	4.47E-06	9.15E-06	0.8753	C47H85O10P	Negative	78.95, 152.99, 331.26
PI(18:1/18:1)	861.5505	0	9.7810	↑	72.7961	1.00E-07	3.07E-07	0.9237	C45H83O13P	Negative	152.99, 223.00, 241.00, 579.30, 597.29
PI(14:0/22:1)	863.5645	1	9.8451	↑	60.5400	8.37E-08	2.88E-07	0.9161	C45H85O13P	Negative	78.96, 152.99, 241.01
PI(16:0/22:3)	887.5644	1	8.3686	↑	56.9735	6.90E-13	9.89E-12	0.7633	C47H85O13P	Negative	78.95, 152.99, 241.01, 553.24
Inosine	267.07301	1	0.4075	↓	0.1724	2.68E-03	3.11E-03	0.7984	C10H12N4O5	Negative	135.03, 108.02, 92.03
Palmitic amide	256.2643	3	1.5642	↓	0.1359	4.10E-13	8.82E-12	0.8695	C16H33NO	Positive	158.154, 144.1381, 116.1068, 102.0914, 88.0753, 74.0598, 57.06
Oleamide	282.2804	4	1.6536	↓	0.9586	7.55E-09	3.61E-08	0.8403	C18H35NO	Positive	265.25, 247.242, 135.117, 97.102, 83.0853, 69.07
Stearamide	284.2957	3	2.21027	↓	0.8763	1.03E-14	4.43E-13	0.8648	C18H37NO	Positive	228.23, 200.20, 186.18, 172.17, 144.13, 116.10, 102.09, 88.07, 74.06
13Z-Docosenamide	338.3427	2	8.8864	↓	0.1942	1.94E-10	1.39E-09	0.8726	C22H43NO	Positive	321.3156, 303.3065, 149.1326, 135.117, 97.10, 83.08, 69.07
3-Deoxyvitamin D3	369.3527	3	12.7020	↑	14.4965	1.54E-06	3.90E-06	0.8619	C27H44	Positive	287.26, 233.22, 215.18, 161.13, 147.11, 133.10, 109.10, 81.06, 67.05
PC(16:0/0:0)	496.3409	2	5.6375	↑	91.6590	1.93E-02	1.98E-02	0.7051	C24H50NO7P	Positive	478.32, 184.07, 166.06, 125.00, 104.10, 86.09, 60.08
Glu Phe Arg Trp	637.3067	3	1.2381	↓	0.2823	7.91E-06	1.48E-05	0.866	C31H40N8O7	Positive	83.05, 147.11, 163.01, 239.04, 337.02, 393.07, 469.12, 525.17, 581.23
PC(14:0/18:2)	730.5416	4	8.9420	↑	286.1312	9.26E-10	5.69E-09	0.9312	C40H76NO8P	Positive	184.07, 242.11, 285.24
PC(18:1/14:0)	732.5552	1	7.6818	↑	4.9261	2.68E-12	2.53E-11	0.8275	C40H78NO8P	Positive	86.09, 184.07, 549.48
PC(16:0/20:5)	780.5545	0	8.8476	↑	40.7199	6.95E-08	2.72E-07	0.9161	C44H78NO8P	Positive	88.11, 184.07, 255.21, 393.24
PC(16:0/20:4)	782.5708	1	6.7811	↑	6.6011	1.17E-02	1.23E-02	0.6482	C44H80NO8P	Positive	86,09, 184.0, 125.00, 258.10, 313.27, 419.2485, 478.3295, 496.34, 526.32, 599.5016
PC(16:0/22:6)	806.5709	1	7.2133	↑	284.1474	1.52E-03	1.92E-03	0.7885	C46H80NO8P	Positive	184.07, 267.21, 478.32, 550.32, 623.50
PC(18:0/20:2)	814.6337	2	9.9895	↑	10.9063	3.56E-04	5.10E-04	0.7925	C46H88NO8P	Positive	184.07, 263.27, 341.30, 508.37
PC(18:1/22:6)	832.5861	1	9.8159	↑	9.9810	8.71E-08	2.88E-07	0.8922	C48H82NO8P	Positive	184.07, 522,35, 568,33

Few studies have used high-throughput approaches to study the changes in metabolomic and lipidomic profiles that underscore EC progression. Hence, in order to understand the metabolic phenotype associated with EC development, we interrogated profile differences in 29 tumor tissues restricted to the uterine cavity (FIGO stages I and II) compared to 10 tumors showing lymph node dissemination (FIGO stage III). We found a set of features to be significantly dysregulated (adjusted p-value < 0.05) among the different FIGO stages (Supplementary Table 2). The PCA plots showing the segregation of the groups and the heat map showing the expression of several features in normal endometrium and in different FIGO stages of the tumor are represented in Fig. 1B and C. A subset of seven metabolites was verified by MS/MS (Table 3). The dysregulation observed in lipids (two PCs and three PEs) in tumor tissues compared to controls was significant along tumor progression. Additionally, we observed that arachidonic acid and UDP-N-acetyl-D-galactosamine are important contributors in the tumor progression, as they appeared to be upregulated in advanced compared to early FIGO stages (Supplementary Figure 3).

**Table 3:** Potential biomarkers confirmed using tandem mass spectrometry. List of metabolites that showed significant alterations in early vs late stages of EC. (*m/z* = mass/charge; ppm = parts per million; RT = retention time; p-v = p-value; FC = fold change; FDR = false discovery rate; AUC = area under the curve).

**Metabolite**	***m/z***	**Mass error (ppm)**	**RT (min)**	**Early vs late EC stages**	**FC (early/late)**	**p-value**	**FDR**	**AUC**	**Formula**	**Ionization mode**	**Column**	**Major CID fragments**
Arachidonic Acid	303.2325	1	6.8246	↓	0.1402	6.24E-03	8.74E-03	0.778	C20H32O2	Negative	C18_BEH	259.25, 216.99, 205.19
PC(16:0/20:5)	780.5545	0	8.8476	↓	0.3311	3.13E-03	5.48E-03	0.750	C44H78NO8P	Positive	C18_BEH	88.11, 184.07, 255.21, 393.24
PC(16:0/22:6)	806.5709	1	9.0460	↑	3.0814	7.86E-04	5.48E-03	0.807	C46H80NO8P	Positive	C18_BEH	184.07, 267.21, 478.32, 550.32, 623.50
PE(16:0/22:6)	762.5106	3	9.0937	↑	2.0349	8.36E-03	9.75E-03	0.752	C43H74NO8P	Negative	C18_BEH	140.01, 255.23, 283.24, 327.23, 452.27
PE(18:1/22:6)	788.5258	2	9.1145	↑	15.2799	2.2E-02	2.20E-02	0.676	C45H76NO8P	Negative	CSH and C18_BEH	78.95 140.01, 152.99, 281.24, 283.26, 327.23, 460.26, 478.29, 506.26
PE(22:6/P-18:1)	772.5278	1	9.3001	↓	0.4065	1.86E-03	5.48E-03	0.800	C45H76NO7P	Negative	CSH and C18_BEH	140.01, 283.24, 327.23, 444.28, 462.29
UDP-N-acetyl-D-galactosamine	606.0739	0	0.5454	↓	0.3765	2.97E-02	5.48E-03	0.688	C17H27N3O17P2	Negative	C18_BEH	111.0222, 158.92, 176.93, 282.04, 362.00, 385.00

### Increased expression of ADAR family of enzymes in human EC tumors

Among the metabolites identified in EC tissues, we were particularly interested in the dysregulation of nucleoside inosine since the relative abundance of this metabolite was significantly higher in EC tumors. To our knowledge, the underlying impact of alterations of endogenous levels of inosine has never been investigated in EC. Dysregulated levels of adenosine and inosine (A/I ratio) can be attributed to the modulation of the A-to-I editing pathway^24^. Consequently, we studied the status of this pathway in EC by analyzing the expression level of members of the A-to-I editing enzymes family (ADAR1 and ADAR2) in three independent sets of samples by immunohistochemistry (IHQ) (Table 4). The first set included the evaluation of 20 EEC samples and their corresponding paired healthy tissues; the second set included 36 EEC tumors diagnosed at different histological grades (low grade, n = 12; intermediate grade, n = 13; high grade, n = 11); and the third set allowed to differentiate between histological subtypes, and so, 78 EEC and 29 NEEC tissue samples were included.

**Table 4:** Clinical and histological information of patients included in the validation set.

		Cohort 1			Cohort 2			Cohort 3		
		EEC (n = 20)	NEEC (n = 0)	Control (n = 20)	EEC (n = 36)	NEEC (n = 0)	Control (n = 0)	EEC (n = 78)	NEEC (n = 29)	Control (n = 0)
Age	> 50	20	—	20	36	—	—	78	29	—
	< 50	—	—	—	——	—	—	—	—	—
Collection center	VHUH	20	—	20	7	—	—	78	29	—
	Lleida	—	—	—	13	—	—	—	—	—
	Santiago	—	—	—	6	—	—	—	—	—
	Virgen Rocío	—	—	—	8	—	—	—	—	—
	MD Anderson	—	—	—	2	—	—	—	—	—
Uterine condition	Pre-menopausal	—	—	—	—	—	—	—	—	—
	Post-menopausal	20	—	20	36	—	—	78	29	—
FIGO stage	IA	10	—	—	13	—	—	17	7	—
	IB	6	—	—	10	—	—	31	5	—
	II	2	—	—	5	—	—	20	4	—
	III	2	—	—	8	—	—	7	11	—
	IV	—	—	—	—	—	—	3	2	—
Histologic grade	G1	4	—	—	12	—	—	13	0	
	G2	7	—	—	13	—	—	33	2	
	G3	9	—	—	11	—	—	32	27	
Format	TMA	—	—	—	36	—	—	78	29	—
	Individual slide	20	—	20	—	—	—	—	—	—

**Figure 2:**
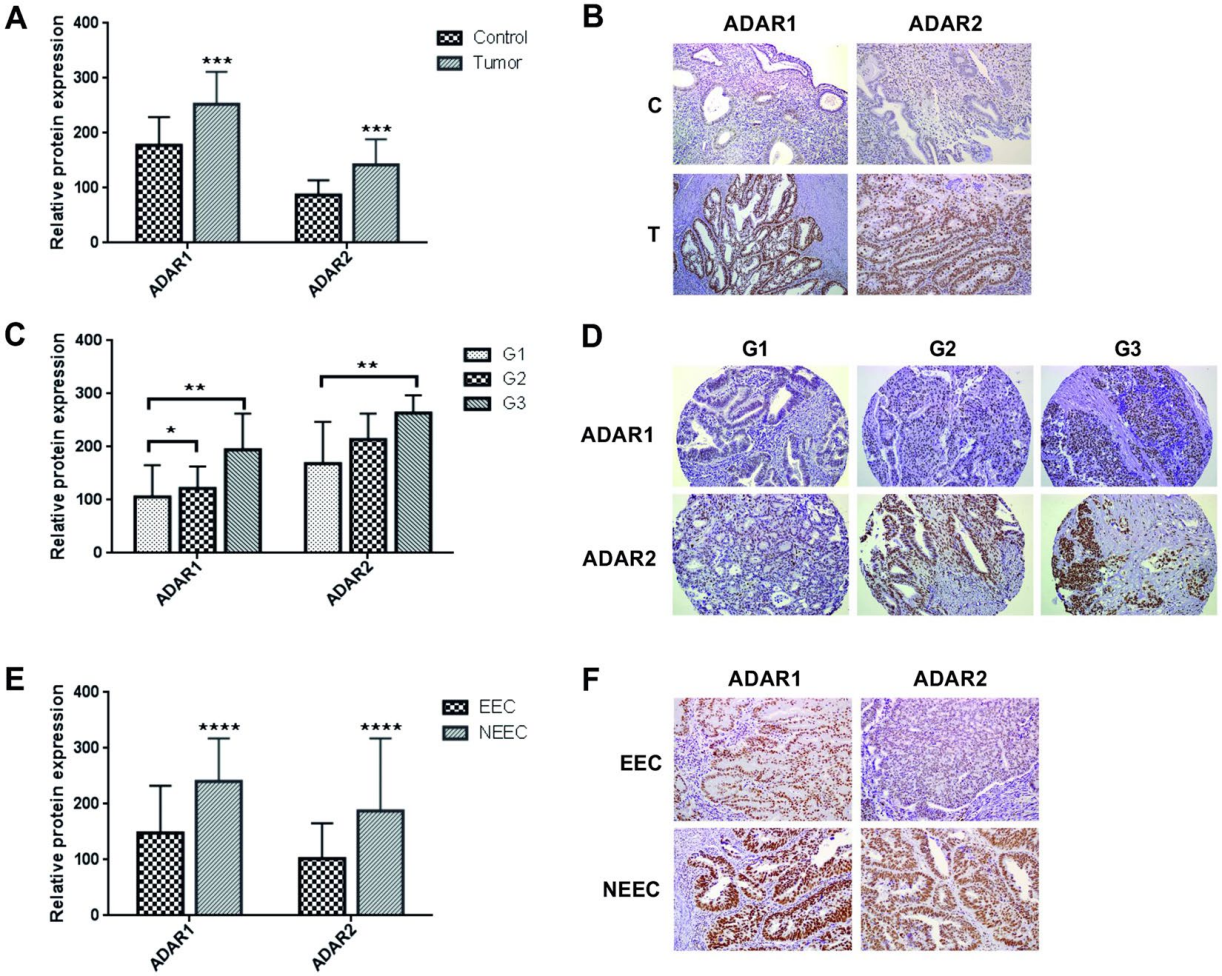
ADAR1 and ADAR2 proteins are overexpressed in EC. Panel A. ADAR1 and ADAR2 expression in EC tumors (T) compared to the paired control (C) tissues. Relative protein expression of each enzyme is plotted in the bar graphs. Panel B. Example of ADAR1 and ADAR2 staining levels in a matched EC tissue with the corresponding paired control. Panel C. ADAR1 and ADAR2 expression in EC tissues significantly correlate with the tumor grade. Panel D. Example of ADAR1 and ADAR2 staining levels in a set of 3 different EC tumor grades slides (grade 1, 2 and 3). Panel E. ADAR1 and ADAR2 levels are significantly increased in NEEC compared to EEC tumors. Panel F. Example of ADARs staining in EEC and NEEC tumors.

Although ADAR1 and ADAR2 staining was clearly localized in the nucleus of epithelial, stromal, and endothelial cells, we specifically analyzed the staining of the epithelial tumor cells (Fig. 2B,D,F and Supplementary Figure 4B). Interestingly, no staining was observed in cells undergoing mitosis (Supplementary Figure 4A). Our results demonstrated that ADAR1 and ADAR2 were both significantly increased in tumor samples compared to healthy endometrial tissue (Fig. 2A), confirming that the RNA editing pathway is activated in EC carcinogenesis. ROC analysis for ADAR1 and ADAR2 expression yielded an AUC of 0.79 and 0.90 respectively, emphasizing the differences observed between control and EC samples (Supplementary Figure 5). Moreover, ADAR expression increased progressively with the presence of poor prognostic factors, such as tumors presenting high grade or a NEEC histology (Fig. 2C and E). These data suggested for the first time an activation of the RNA editing pathway in patients with EC, which positively correlates with aggressive disease, resulting in a dysregulation of the nucleosides/nucleotides balance in the tumor tissue.

### Inhibition of ADAR2 reduces viability, increases apoptosis and reduces migration capabilities in human EC cell lines

**Figure 3:**
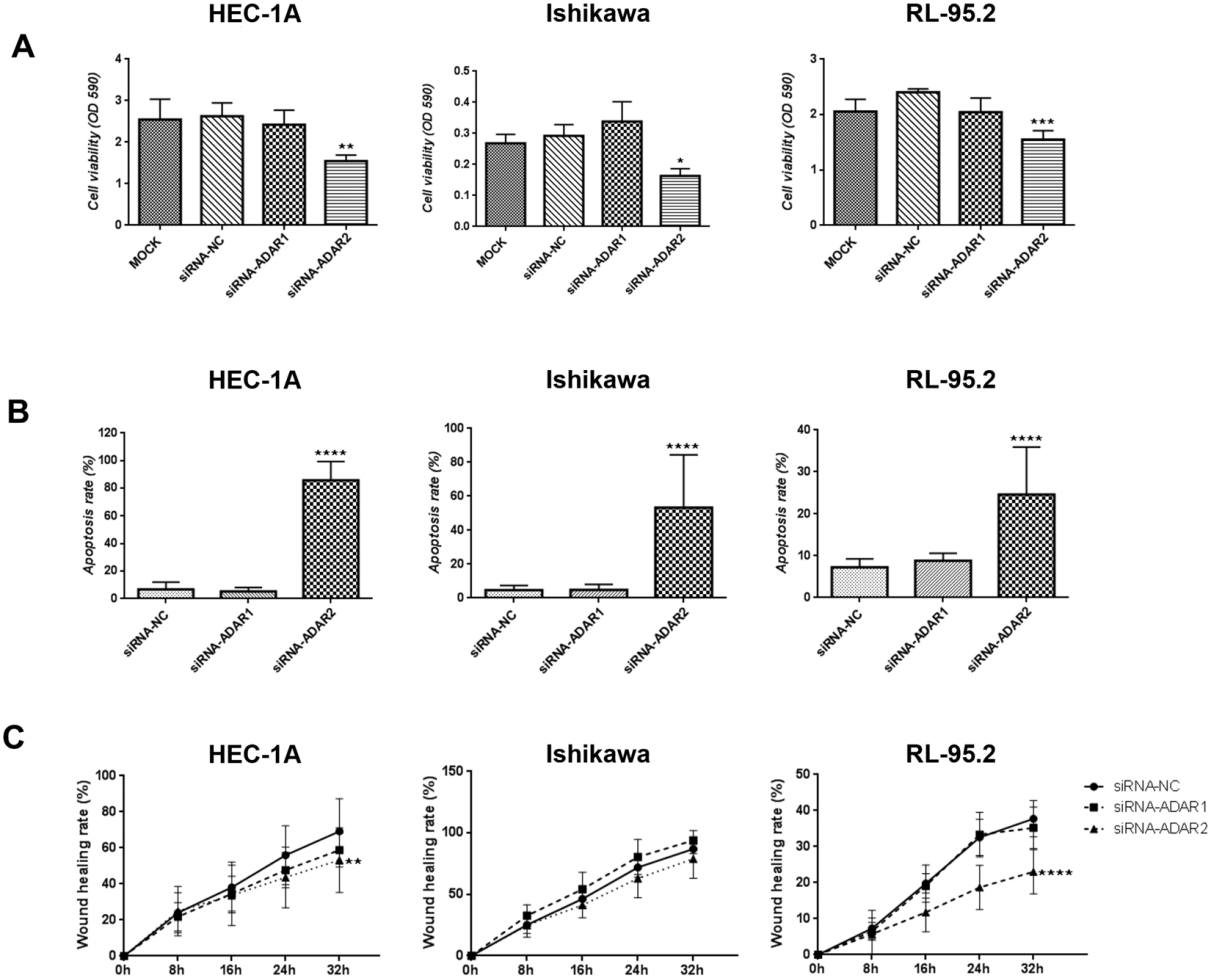
Functional assays revealing that ADAR2 presents oncogenic functions *in vitro*. HEC-1A, Ishikawa and RL95-2 EC cell lines were used for the functional assays. Panel A. Proliferation assay showing a significant decrease in cell viability (OD 590 nm) in the 3 cell lines when inhibiting ADAR2 expression. Panel B. Apoptosis assay showing a significant increase in apoptosis rate when silencing ADAR2. Panel C. Wound healing assay indicating a significant decrease in HEC-1A and RL95-2 migration capabilities (% of wound healing) when treating cells with siRNA-ADAR2. No significant changes were seen when inhibiting ADAR1.

Next, in order to determine the possible role of the RNA editing pathway in EC progression, we transiently silenced the expression of the ADAR1 and ADAR2 enzymes in three EC cell lines (HEC-1A, Ishikawa and RL95-2). Transfection efficiency was determined by comparing each transfection against the corresponding negative control (NC) (Supplementary Figure 6). Gene silencing for both enzymes was confirmed by western blot analysis (WB) and immunofluorescence (IF) after 96 h of transfection (Supplementary Figure 7), time point in which the modulation of cell growth and cell viability in EC cell lines was assessed. The inhibition of ADAR2, but not ADAR1, resulted in a significant reduction of cell viability and proliferation compared to controls in the three cell lines used in this study (Fig. 3A). Furthermore, the ratio of apoptotic cells significantly increased in the three cell lines upon silencing of ADAR2. Similarly, proliferation assay performed using cells treated with siRNA-ADAR1, did not result in significant changes in apoptosis (Fig. 3B). Finally, the migration capabilities of the EC cell lines were interrogated after inhibiting ADAR1 and ADAR2 in a wound healing assay. Knockdown of ADAR2 expression resulted in significant reduction of the migration rate in HEC-1A and RL95-2 cell lines. Remarkably, similar changes were not observed when the same cell lines were treated with siRNA-ADAR1 (Fig. 3C).

In order to further confirm the functional role of ADAR2 in EC cell lines, we also conducted functional assays silencing ADAR2 expression with two new and different siRNAs (siRNA-ADAR2_B and siRNA-ADAR2_C), independent from the siRNA-ADAR2 used in Fig. 3. We corroborate a significant decrease of cell viability, a significant increase in apoptosis rate and a significant decrease of wound healing rate of the 3 EC cell lines induced by the silencing of ADAR2 expression (Supplementary Figure 8). These results clearly demonstrate that changes in the expression of ADAR2 leads to significant changes in the aggressive behavior of EC cell lines, specifically on cell viability and apoptosis, and cell migration capabilities of EC cell lines.

## Discussion

**Figure 4:**
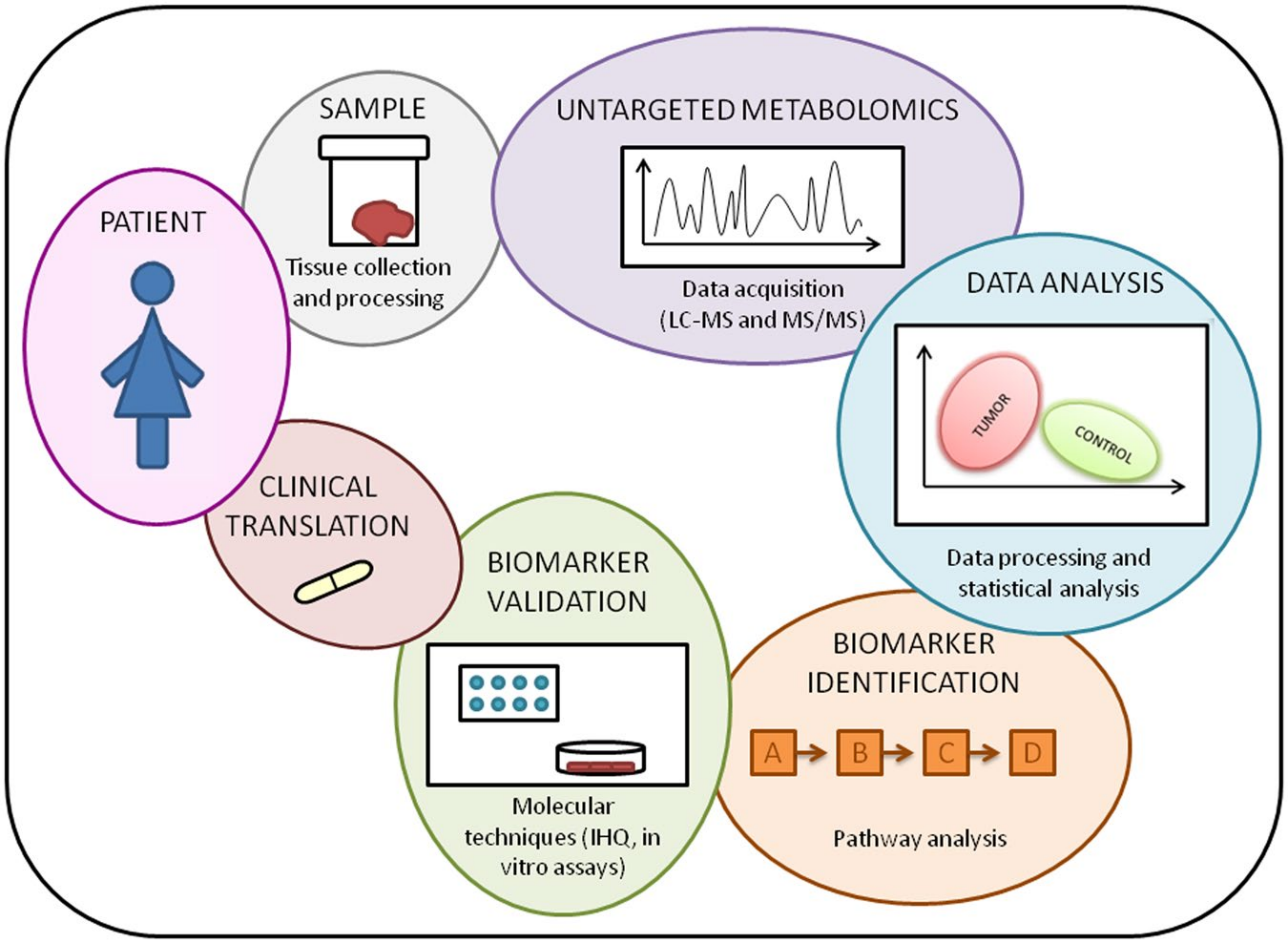
Project workflow summary.

Recently, few studies have reported alterations in the metabolomic or proteomic phenotype of EC in serum^25^, urine^26^ or in other sample types^27, 28^, underscoring the clinical translational relevance of these technologies in furthering the personalized medicine initiative. In this study, we used a global metabolomics profiling approach in order to understand the metabolic changes that take place in EC carcinogenesis and during tumor progression (Fig. 4).

Our study reveals an array of metabolites dysregulated in EC, some of which have been previously described in endometrial carcinogenesis. Glycerophospholipid class of metabolites was found to be upregulated in tumor tissues, including PCs, PEs, and PIs. Lipid biosynthesis and catabolism is known to be altered in several diseases, including cancer^28–32^. Consistent with our findings, Trousil *et al*.^28^ also observed an increase (up to 70%) in PC levels in EC tissues. We also observed a downregulation of the acylamido analogs of endocannabinoids such as palmitamide, stearamide and oleamide in EC. This downregulation has been previously reported^33^. We also observed a decreased proportion of picolinic acid in tumor samples. Picolinic acid and quinolinic acid are the end products of the Kynurenine pathway and have been shown to have anti-tumoral and pro-tumoral activity respectively^34^. Moreover, the activity of one of the main enzymes of the pathway, indoleamine 2,3-dioxygenase (IDO) has also been studied in EC^35^. Decreased levels of Glu Phe Arg Trp and inosine as well as upregulation of 3-Deoxyvitamin D3 and UDP-N-acetyl-D-galactosamine were also found in our EC tumor sample set compared to control tissues. We also analyzed metabolomic changes that underscore tumor progression. Our data suggest significant changes in the lipidome including PC (16:0/20:5), PC (16:0/22:6), PE (16:0/22:6), PE (22:6/P-18:1) and PE (18:1/22:6) at different stages of cancer progression, as well as a significant increase in the levels of UDP-N-acetyl-D-galactosamine and arachidonic acid at advanced stages of EC. Further studies would be needed to fully understand the scope and impact of these alterations in cancer progression.

The dysregulation of inosine was further studied to dissect the functional implications of the RNA editing pathway in EC. The dysregulation of inosine is indicative of a possible imbalance in the I/A ratio, which was also reported by Trousil *et al*.^28^ in studies with EC tissue compared to normal endometrium. The A-to-I conversion is the most common type of RNA editing found in mammals mediated by the ADAR enzymes. Although, to date, the RNA editing pathway and the expression and function of the ADAR gene family has not been interrogated in EC, it has been reported that the expression of ADAR enzymes is upregulated in many cancers^18, 19^, including breast^17^ and esophageal squamous cell carcinoma^36, 37^. The ADAR family is comprised of three members: ADAR1 and ADAR2, that are present in most human tissues; and ADAR3, that is brain specific^23^. Changes in editing frequencies have been described in other diseases including prostate, lung, kidney and testis tumors while reduced RNA levels of ADAR1, ADAR2 and ADAR3 have been observed in brain tumors^38, 39^. ADAR enzymes are also involved in physiological events such neuronal development, immune response, cell response to viruses and regulation of miRNA expression among others^40, 41^.

Hence, we asked if the expression of ADAR enzymes had any correlation with EC initiation and progression. Our findings not only elucidate ADAR1 and ADAR2 enzymes to be significantly upregulated in EC tumor tissues compared to healthy endometrium, but also demonstrate a significant correlation between ADARs expression and the malignancy of the tumor. We found that the ADARs expression increased progressively with tumor grade. More importantly, our data showed a significant increase in the expression of ADAR1 and ADAR2 in the most aggressive subtype of EC, the NEEC, that have the worst predicted survival^3^.

The role of the RNA editing pathway in EC was further investigated by knocking-down the expression of ADAR1 and ADAR2 in HEC-1A, RL95-2 and Ishikawa EC lines. Our results demonstrate the impact of decreased ADAR2 expression on an array of cellular functions in EC cell lines including a significant decrease of cell proliferation and viability, increased apoptosis rate, and reduced migration capabilities *in vitro*. Similar to our observations, silencing of ADARs in breast cancer cell lines led to less cell proliferation and more apoptosis^17^ and overexpression of editing enzymes accelerated growth rate and colony formation in esophageal squamous cell carcinoma *in vitro* ^36^. Taken together, our results strongly suggest, for the first time, that the RNA editing gene family, specifically ADAR2, may play an important role promoting EC carcinogenesis.

In conclusion, a global molecular profiling approach using high resolution mass spectrometry has been useful, not only to describe changes in the metabolome and lipidome in human endometrial carcinogenesis and EC progression but also led to the discovery of an important alteration of the RNA editing pathway in EC. We further demonstrated the role of this pathway in proliferation and viability, apoptosis, and migration of EC cells, leading us to conclude that the activation of the RNA editing pathway is an oncogenic process in EC. This study opens several avenues for further investigations of ADAR2 as possible target for the development of therapeutic approaches for the treatment of EC patients.

## Methods

### Patients and tissue collection

#### Discovery and verification phase

A total of 56 women (39 diagnosed with EC and 17 non-EC patients) were recruited at Vall Hebron University Hospital (VHUH) in Barcelona, Spain. All patients participating signed an informed consent and the study was approved by the Clinical Research Ethics Committee (CREC) at the VHUH (approval number: PR_AMI_50–2012). Endometrial tissues were collected at the histopathology department of VHUH after the surgical intervention. Each tissue piece (about 5 to 10 mg) was placed in a sterile and separate container, properly labeled and stored at −80 °C immediately. A description of the clinical and pathological characteristics of the tumors is detailed in Table 1. Inclusion criteria: post-menopausal women, > 50 years, no previous treatment for pelvic gynecological cancer, negative for HIV and hepatitis viruses.

#### Validation phase

Patients split in 3 new cohorts were enrolled in VHUH or in University Hospital Arnau de Vilanova of Lleida following the approval of the CREC at each participating institution. Tissue samples were embedded in paraffin block for individual slides or tissue microarray (TMA) construction in VHUH. A description of the clinical and pathological characteristics of the tissues is detailed in Table 4.

### Tissue metabolomics using Ultra Performance Liquid Chromatography coupled to Quadrupole Time-Of-Flight Mass Spectrometry (UPLC-QTOF-MS)

Reagents and chemicals: Solvents using chloroform, ACN, water and methanol were purchased from Fisher Optima grade, Fisher Scientific (New Jersey, USA). High purity formic acid (99%) was purchased from Thermo-Scientific (Rockford, IL, USA). Ammonium formate, debrisoquine and 4-nitrobenzoic acid (4-NBA) were purchased from Sigma- Aldrich (St. Louis, MO, USA).

For metabolomics analysis, endometrial tissue samples were prepared following the procedure previously described.^42^ Fresh frozen tissue sections were homogenized on ice using a buffer containing 50% methanol and internal standards (1 mg/ml debrisoquine in distilled water, 1 mg/ml of 4-nitrobenzoic acid in methanol, 10 µg/ml of phosphatidic acid in 50% methanol-water, 0.1 µg/ml of lysophosphatidylcholine in 50% methanol-water). Protein precipitation was done by adding a 1:1 ratio of acetonitrile (ACN). Samples were centrifuged, the supernatant (Supernatant 1) was transferred to a fresh vial and dried under vacuum and the pellet was resuspended in prechilled dichloromethane:methanol (3:1). After sonication and centrifugation, the supernatant (Supernatant 2) was transferred to a fresh vial and dried under vacuum and the pellet was kept for protein estimation. Supernatant 1 and Supernatant 2 were finally resuspended in a buffer containing methanol:ACN:water in a ratio 50:25:25 for LC-MS analysis. The extraction procedure (from aqueous to semi-polar and lastly non-polar solvent) allows the isolation of a wide range of metabolites. In parallel, the pellet was resuspended with RIPA buffer and centrifuged in order to quantify the protein amount using Bradford method^43^. Resuspended pellets (5 µl) were injected onto Acquity UPLC CSH 1.7 µm, 2.1 × 100 mm column (Waters Corp.) or in a BEH C18 1.3 µm, 2.1 × 50 mm column. The mobile phase gradient consisted of ACN/water (60/40) containing 10 mM ammonium formate and 0.1% formic acid (Solvent A) and IPA/ACN (90/10) containing 10 mM ammonium formate and 0.1% formic acid (Solvent B). UPLC separation was performed at a flow rate of 0.4 ml/min for 20 min. Two different gradients were used in order to analyze the lipidome or the metabolome of the sample. MS data acquisition was performed using ESI-QTOF MS within the mass range of 50 to 1200 mass-to-charge ratio (*m/z*) in positive and negative electrospray ionization modes on a SYNAPT G2 Si (Waters Corporation, USA). The capillary voltage used was 3.2 kV and a sampling cone voltage of 30 V in negative mode and 20 V in positive mode. The desolvation gas flow was set to 750 l/h, and the temperature was set to 350 °C. The cone gas flow was 25 l/h, and the source temperature was 120 °C. Accurate mass was maintained by infusion of LockSpray interface with Leucine Enkaphalin (556.2771 [M + H]^+^ and 554.2615 [M − H]^−^). Data were acquired in TOF MS centroid mode and also in continuum mode for the mass range of 50 to 1200 mass-to-charge ratio (*m/z*) with MS scanning at a rate of 0.3 seconds. Total protein concentration for each sample was used to normalize any inconsistencies that would arise during tissue sampling. Subsequently, these data were normalized to internal standard to correct for analytical inconsistencies that could potentially occur during MS batch acquisition.

MS data were pre-processed using the XCMS software^44^. In order to determinate the identification of the metabolites based on the mass and charge, the following databases were used: Human Metabolome Database (www.hmdb.ca), Madison Metabolomics Consortium Database (mmcd.nmrfam.wisc.edu), LIPID MAPS (www.lipidmaps.org), KEGG (www.kegg.jp/kegg), and Metlin (metlin.scripps.edu). Multivariate data analysis was performed using Metaboanalyst 3.0 web tool^45, 46^ and a sub-set of metabolites were verified by tandem mass spectrometry (MS/MS) and using Mass Fragments software (Waters Corp.).

### Immunohistochemistry

A total of three TMAs were constructed as described previously^47^. Paraffin-embedded TMA or individual sections of the samples were mounted on slides, deparaffined and rehydrateted. Antigen retrieval was done during 4 min at 115 °C (pH 6) and subsequently blocked with peroxidase (3%) for 5 min and incubated with the primary antibody for 1 h and 30 min at room temperature in a humidified chamber. After blocking for 30 min with EnVision secondary antibody (Ref. K5007-100 ml, Agilent; Santa Clara, CA, USA) slides were treated with DAB (Ref. K3468, Agilent; Santa Clara, CA, USA) reagent and counterstained with hematoxylin for 30 sec. Finally, a dehytratation step and DPX mounting were performed. Pictures were taken using the Olympus BX41 microscope (20X). The following antibodies were tested: ADAR1 (Ref. AMAb90535, dilution 1:100, Sigma-Aldrich; St. Louis, MO, USA) and ADAR2 (Ref. sc-73409, dilution 1:50, Santa Cruz; Heidelberg, Germany, EU). A pathologist evaluated the expression using two criteria: the intensity of staining (0, no staining; 1, weak intensity; 2, moderate intensity; 3, high intensity) and the percentage of endometrial epithelial stained cells (0–100). The product of the two scores yielded final values on a sacale ranging from 0 to 300.

### Cell lines

The following EC epithelial cell lines were used in this study. HEC-1A (Ref. HTB112, ATCC; Manassas, VA, USA) cell line was cultured in McCoys 5 A medium (Ref. 80014020, ThermoFisher; Waltham, MA, USA); Ishikawa (Ref. 99040201, Sigma-Aldrich; St. Louis, MO, USA) and RL95-2 (CRL-1671, ATCC; Manassas, VA, USA) were cultured in DMEM/F12 (Ref. 11320-033, ThermoFisher; Waltham, MA, USA). Both supplemented with penicillin/streptomycin (1%) and fetal bovine serum (10%). Incubated at 37 °C in a 5% CO2 humidified chamber. Cell lines were morphologically and genetically authenticated and tested for mycoplasma in accordance with AACR guides.

### Western Blot

Protein extraction from cell lines was performed using RIPA buffer (5 nM EDTA, 150 mM NaCl, 1% Triton, 20 nM Tris pH 8 and 1:200 protein inhibitors). After Bradford colorimetric protein quantification samples were run on a SDS/PAGE acrylamide gel for 2 h and transferred to a PVDF membrane. Membranes were blocked for 1 h with 5% milk and incubated with the primary antibodies described above overnight and with the secondary antibodies -Goat anti-rabbit (Ref. P0448, dilution 1:2000, Agilent; Santa Clara, CA, USA) and rabbit anti-mouse (Ref. P0260, dilution 1:2000, Agilent; Santa Clara, CA, USA)- for 1 h at room temperature. Membranes were developed using Immobilon Immobilon Western Chemiluminiscent (Ref. WBKLS0100, Merck Millipore; Billerica, MA, USA). Membranes were finally stained with naphtol blue (Ref. N3393, Sigma-Aldrich; St. Louis, MO, USA) to detect all proteins transferred to the membranes in order to normalize the ADAR protein bands. The following antibodies were tested: ADAR1 (Ref. AMAb90535, dilution 1:100, Sigma-Aldrich; St. Louis, MO, USA) and ADAR2 (Ref. sc-73409, dilution 1:50, Santa Cruz; Heidelberg, Germany, EU).

### Immunofluorescence

Cells were fixed for 10 min 96 h after transfection. Cells were blocked for 15 min with 5% BSA solution in 10 ml of 0.5% PBS-T. They were incubated with the primary antibody for 2 h at room temperature and with the secondary antibody for 45 min at room temperature in a dark chamber. Mounting and DAPI staining were performed with ProLong Gold Antifade reagent with DAPI (Ref. P36931, ThermoFisher; Waltham, MA, USA). Pictures were taken under the fluorescence microscope Nikon Eclipse TE2000-S. Primary antibodies: ADAR1 (Ref. AMAb90535, dilution 1:100, Sigma-Aldrich; St. Louis, MO, USA) and ADAR2 (Ref. sc-73409, dilution 1:50, Santa Cruz; Heidelberg, Germany, EU). Secondary antibody: Alexa Fluor 488 (Ref. A11017, Labeling and detection, ThermoFisher; Waltham, MA, USA).

### Reverse Transfection of siRNAs

Desired number of cells according to the assay (see proliferation, apoptosis and wound healing assay sections for the exact number of cells) was seeded using media without antibiotic supplementation. Transfection mix was prepared in the following proportion: 25 µl of OptiMEM (Ref. 11058021, Invitrogen-ThermoFisher; Waltham, MA, USA), 0.2 µl of lipofectamine (Ref. 11668019, Invitrogen-ThermoFisher; Waltham, MA, USA), and 0.375 µl of siRNA (50 nM) and added in a 1:3 (v/v) to final volume of the well. After 24 h of transfection, media was changed. Pre-designed siRNAs for ADARs were purchased from Sigma-Aldrich: siRNA-ADAR1: SASI Hs01 00115047- GACUAUCUCUUCAAUGUGU; siRNA-ADAR2: SASI Hs01 00237747-GAGUGAUCGUGGCCUUGCA; siRNA-ADAR2_B: SASI Hs01 00222314- GAGUGAUCGUGGCCUUGCA; siRNA-ADAR2_C: SASI_Hs01_00135204- GAGUGAUCGUGGCCUUGCA; siRNA-NC: negative control, BLOCK-it Fluorescent Oligo, for lipid transfection (Ref. 2013, ThermoFisher; Waltham, MA, USA).

### Cell proliferation assay

In order to determine cell proliferation and viability, a total of 4 × 10^3^ cells (HEC-1A); 12 × 10^3^ cells (Ishikawa), and 2 × 10^4^ cells (RL95-2) per well were plated in a 96 well plate (6 replicates per condition). The cell growth rate was measured after 96 h of transfection. Cells were fixed to the plate with glutaraldehide 1% (Ref. G6257, Sigma-Aldrich; St. Louis, MO, USA) for 15 min and stained with Crystal Violet (Ref. C3886, Sigma-Aldrich; St. Louis, MO, USA). After 20 min, cells were washed with H_2_O and Acetic Acid 15% (Ref. 0641, ThermoFisher; Waltham, MA, USA) was added. After 10 min shaking plates, they were read at 590 nm. Three independent experiments were carried out.

### Apoptosis assay

In order to determine cell apoptosis rate, a total of 8 × 10^4^ cells (HEC-1A); 5 × 10^4^ cells (Ishikawa), and 2 × 10^5^ cells (RL95-2) per well were seed in a p24 (8 replicates per condition). After 96 h of transfection, cells were stained with Hoechst (1:1000, Ref. H6024, Sigma-Aldrich; St. Louis, MO, USA) and after 24 h pictures were taken under the fluorescence microscope Nikon Eclipse TE2000-S. The number of nucleus with condensed chromatin and strongly staining of the DNA were quantified as apoptotic cells and divided by the total number of nucleus. Three independent experiments were carried out (4 camps per well were counted).

### Wound healing assay

To evaluate cell migration capabilities, a total of 8 × 10^4^ cells (HEC-1A); 5 × 10^4^ cells (Ishikawa), and 2 × 10^5^ cells (RL95-2) per well were seed in a p24 (3 replicates per condition). Wound was generated 72 h post-transfection. Pictures were taken every 8 h using an Olympus FSX100 microscope. Wound healing area was measured using Image J software at the different time points. Three experiments were carried out independently.

All experiments were performed in accordance with the relevant guidelines and regulations.

### Statistical analysis

The SPSS statistical package version 23 for Windows® and the GraphPad Prism version 6 were used to perform the statistical analyses and ROC analysis. Each value represents the mean of at least 3 replicates with the corresponding standard deviation. We analyzed the normality of each data set and we used, according to the sample distribution, parametric or no parametric tests. For the IHQ analysis, a Wilcoxon signed rank test was used to compare tumors from controls; and a Kruskal-Wallis and Mann-Whitney tests were applied when comparing protein expression according to grades and histological subtypes, respectively. For the functional analysis, means of the different groups were compared by Kruskal-Wallis followed by Dunn’s multiple comparisons test (in case of significance). We considered significant p-values < 0.05. (*p-v < 0.05; **p-v < 0.01; ***p-v < 0.001; ****p-v: 0.0001).

## Electronic supplementary material

Supplementary information
