# Supplementary material for: Metabolomic and Lipidomic Profiling Identifies The Role of the RNA Editing Pathway in Endometrial Carcinogenesis
Source: Sci Rep. 2017 Aug 18;7:8803. doi: 10.1038/s41598-017-09169-2 (PMC5562852; doi:10.1038/s41598-017-09169-2)

METABOLOMIC AND LIPIDOMIC PROFILING IDENTIFIES THE ROLE OF THE RNA EDITING PATHWAY IN ENDOMETRIAL CARCINOGENESIS

Tatiana Altadill[1], Tyrone M. Dowdy[2], Kirandeep Gill[2], Armando Reques[3], Smrithi S Menon[2], Cristian P Moiola[1], Carlos Lopez-Gil[1], Eva Coll[1], Xavier Matias-Guiu[4], Silvia Cabrera[5], Angel Garcia[3], Jaume Reventos[1,6], Stephen W Byers[2,7], Antonio Gil-Moreno[3,5], Amrita K Cheema[2,7]* and Eva Colas[1]*

1 Biomedical Research Group in Gynecology, Vall Hebron Research Institute (VHIR), Universitat Autònoma de Barcelona, Barcelona, Spain. CIBERONC.

2 Department of Oncology, Georgetown University Medical Center. Washington D.C. USA.

3 Pathology Department, Vall Hebron University Hospital, Barcelona, Spain.

4 Pathological Oncology Group and Pathology Department, University Hospital Arnau de Vilanova, and University Hospital Bellvitge, IRBLLEIDA and Idibell, University of Lleida. CIBERONC.

5 Gynecological Oncology Department, Vall Hebron University Hospital, Barcelona, Spain.

6 Basic Sciences Department, International University of Catalonia, Barcelona, Spain. CIBERONC.

7 Department of Biochemistry Molecular and Cellular Biology, Georgetown-Lombardi Comprehensive Cancer Center.

*These authors contribute equally to the work.

CORRESPONDING AUTHOR (*)

Eva Colas Ortega. Biomedical Research Group in Gynecology, Vall Hebron Research Institute (VHIR), Pg. Vall Hebron 119-129. 08035 Barcelona (Spain). Phone: 0034-93.489.31.23 Email: eva.colas@vhir.org

SUPPLEMENTARY TABLES

Supplementary Table 1. List of metabolites putatively identified (based on accurate mass) in the discovery set that showed significant changes in their relative abundance in EC tissues compared to the controls. The identity of a sub-set of metabolites confirmed, using tandem mass spectrometry and matching the fragmentation pattern to a reference standard, are listed in Table 2.

Metabolite	*m/z*	Mass error (ppm)	RT (min)	FC (T/N)	p-value	Formula	Mode
Picolinic acid	122.0245	2	0.650	0.183	3.07E-03	C6H5NO2	Negative
Palmitic amide	256.2644	3	1.564	0.014	4.10E-13	C16H33NO	Positive
His Ile	267.1458	1	5.165	41.789	2.49E-02	C12H20N4O3	Negative
Linoleic acid	279.2320	3	6.905	3.282	2.33E-02	C18H32O2	Negative
Oleamide	282.2804	4	1.654	0.010	7.55E-09	C18H35NO	Positive
Stearamide	284.2957	3	2.210	0.088	1.03E-14	C18H37NO	Positive
3-Deoxyvitamin D3	369.3527	3	12.702	14.497	1.54E-06	C27H44	Positive
15beta-Hydroxy-7alpha-mercapto-pregn-4-ene-3,20-dione 7-acetate	403.1940	2	6.526	6.063	2.37E-03	C23H32O4S	Negative
Gly His Pro Val	409.2184	2	0.780	0.112	9.39E-06	C18H28N6O5	Positive
Lys Met His	415.2128	1	4.775	133.472	1.56E-05	C17H30N6O4S1	Positive
Gly Pro Arg Ser	416.2247	1	0.733	6.407	8.96E-11	C16H29N7O6	Positive
Ala Lys Asn Ser	417.2103	0	6.955	186.790	1.38E-03	C16H30N6O7	Negative
Ala Ala Lys Met	418.2134	0	6.329	23.056	1.16E-03	C17H33N5O5S	Negative
Ala Ala Ser Trp	432.1879	2	4.760	0.115	3.62E-03	C20H27N5O6	Negative
PE(P-16:0/0:0)	436.2827	1	5.814	8.455	1.82E-03	C21H44NO6P	Negative
Trp Met Asp	451.1664	3	0.652	0.370	7.81E-03	C20H26N4O6S1	Positive
Cys Lys Thr Cys	454.1790	0	0.713	52.837	1.28E-03	C16H31N5O6S2	Positive
1-O-alpha-D-glucopyranosyl	477.3807	4	0.733	129.167	1.13E-04	C26H52O7	Positive
PE(18:0/0:0)	480.3092	0	6.305	11.485	7.95E-04	C23H48NO7P	Negative
PC(16:0/0:0)	496.3409	2	5.637	91.659	1.93E-02	C24H50NO7P	Positive
Ala Glu Lys Arg	501.2812	4	5.336	0.386	3.21E-03	C20H38N8O7	Negative
Thr Thr Gly Leu Ile	502.2902	3	5.493	15.121	8.04E-04	C22H41N5O8	Negative
Ile Ile Met Arg	530.3152	4	6.199	26.818	3.44E-06	C23H45N7O5S	Negative
Glu Lys Lys Lys	532.3430	4	5.768	16.779	9.04E-03	C23H45N7O7	Positive
Asp His Lys Arg	553.2860	1	5.187	37.104	4.69E-04	C22H38N10O7	Negative
His Ile Met Arg	554.2877	0	5.143	10.703	4.84E-03	C23H41N9O5S	Negative
PG(22:6/0:0)	555.2716	2	5.656	5.286	4.68E-03	C28H45O9P	Negative
Arg Arg Met Leu	573.3305	0	6.330	49.118	4.38E-02	C23H46N10O5S	Negative
Lys Thr Glu Lys Ala	574.3226	3	6.329	22.446	1.27E-03	C24H45N7O9	Negative
Lys Lys Tyr Tyr	599.3197	0	6.653	50.352	1.79E-06	C30H44N6O7	Negative
UDP-N-acetyl-D-galactosamine	606.0739	0	0.545	59.891	4.41E-08	C17H27N3O17P2	Negative
His Gln Tyr Tyr	608.2452	3	9.007	0.014	3,96E-27	C29H35N7O8	Negative
Lys Thr Trp Trp	620.3220	4	6.141	34.551	2.34E-02	C32H41N7O6	Positive
Glu Phe Arg Trp	637.3067	3	1.238	0.028	7.91E-06	C31H40N8O7	Positive
SM(d18:1/14:0)	675.5442	1	8.327	6.491	2.30E-03	C37H75N2O6P	Positive
PE(16:1/P-18:1)	698.5116	2	9.570	2.963	9.80E-03	C39H74NO7P	Negative

SM(d16:1/18:1)	701.5599	0	8.436	4.410	8.92E-04	C39H77N2O6P	Positive
PA(O-16:0/21:0)	705.5816	3	7.054	0.132	8.23E-05	C40H81O7P	Positive
PE(16:0/17:0)	706.5414	4	7.300	382.658	4.95E-08	C38H76NO8P	Positive
PE(16:0/18:2)	714.5078	0	9.402	14.459	1.16E-04	C39H74NO8P	Negative
PE(18:1/16:0)	716.5231	0	9.277	20.105	6.31E-04	C39H76NO8P	Negative
PE(18:4/P-18:1)	720.4964	1	9.082	12.357	1.50E-04	C41H72NO7P	Negative
PC(14:0/18:2)	730.5389	1	8.942	58.473	4.66E-11	C40H76NO8P	Positive
SM(d18:1/18:0)	731.6070	1	8.726	0.016	4.59E-12	C41H83N2O6P	Positive
PC(18:1/14:0)	732.5552	1	7.682	4.926	2.68E-12	C40H78NO8P	Positive
PE(18:0/18:3)	740.5213	3	9.459	2.486	8.10E-07	C41H76NO8P	Negative
PE(18:1/18:1)	742.5388	0	9.428	86.813	2.11E-06	C41H78NO8P	Negative
PE(18:0/18:1)	746.5703	1	8.345	100.242	4.96E-08	C41H80NO8P	Positive
PE(O-16:0/22:5)	752.5607	2	9.745	0.139	5.42E-09	C43H78NO7P	Positive
PG(13:0/22:6)	753.4738	4	0.690	11.581	4.54E-05	C41H69O10P	Positive
SM(d18:1/20:0)	759.6383	1	9.807	0.116	1.21E-05	C43H87N2O6P	Positive
PG(O-16:0/20:1)	761.5677	3	9.188	2.404	3.40E-04	C42H83O9P	Negative
PE(16:0/22:6)	762.5106	3	9.094	2.526	2.95E-04	C43H74NO8P	Negative
PC(15:0/20:5)	764.5268	4	8.250	16.476	1.33E-05	C43H76NO8P	Negative
PE(22:6/P-18:1)	772.5278	1	9.300	0.231	2.17E-03	C45H76NO7P	Negative
PS(13:0/22:1)	774.5303	1	8.903	76.552	2.09E-09	C41H78NO10P	Negative
PA(20:2)/22:4)	775.5311	3	8.895	5.340	1.97E-12	C45H77O8P	Negative
PS(13:0/22:0)	776.5439	1	7.668	161.392	9.88E-14	C41H80NO10P	Negative
PA(20:1/22:4)	777.5470	3	7.668	12.800	1.24E-12	C45H79O8P	Negative
PC(16:0/20:4)	782.5708	1	6.781	6.601	1.17E-02	C44H80NO8P	Positive
SM(d18:1/22:0)	787.6701	1	10.615	0.017	1.75E-09	C45H91N2O6P	Positive
PE(18:1/22:6)	788.5258	2	9.115	8.998	9.22E-05	C45H76NO8P	Negative
PS(18:0/18:1)	790.5608	2	9.072	0.067	1.22E-09	C42H80NO10P	Positive
PS(15:0/22:2)	800.5437	1	8.995	5.224	5.79E-07	C43H80NO10P	Negative
PC(16:0/22:6)	806.5709	1	7.213	284.147	1.52E-03	C46H80NO8P	Positive
SM(d18:2/24:1)	811.6659	3	9.929	0.155	1.97E-10	C47H91N2O6P	Positive
PC(18:0/20:2)	814.6337	2	9.990	10.906	3.56E-04	C46H88NO8P	Positive
SM(d18:1/24:0)	815.7016	1	11.266	0.146	1.47E-05	C47H95N2O6P	Positive
PE(20:1/22:6)	816.5526	2	8.940	0.052	2.94E-12	C47H80NO8P	Negative
PS(17:0/22:4)	824.5433	1	8.838	5.989	9.13E-07	C45H80NO10P	Negative
PS(17:0/22:2)	828.5754	0	8.269	2.310	8.09E-05	C45H84NO10P	Negative
PC(18:1/22:6)	832.5861	1	9.082	9.981	8.71E-08	C48H82NO8P	Positive
PI(16:0/18:1)	835.5348	0	9.764	20.423	5.08E-06	C43H81O13P	Negative
PG(19:0/22:4)	839.5768	4	9.803	0.424	4.47E-06	C47H85O10P	Negative
Galbeta1-4Glcbeta-Cer(d18:1/16:0)	860.6069	4	8.906	4.897	1.22E-05	C46H87NO13	Negative
PI(18:1/18:1)	861.5505	0	9.781	72.796	1.00E-07	C45H83O13P	Negative
PI(14:0/22:1)	863.5645	1	9.845	60.540	8.37E-08	C45H85O13P	Negative
PI(16:0/22:3)	887.5644	1	8.369	56.974	6.90E-13	C47H85O13P	Negative
Inosine	267.07301	1	0.407	0.172	2.68E-03	C10H12N4O5	Negative
PC(16:0/20:5)	780.55447	0	8.848	40.720	6.95E-08	C44H78NO8P	Positive

Supplementary Table 2. List of features that showed significant dysregulation in the different FIGO stages of EC tissues that were interrogated in this study. The same input mass or metabolite can appear more than once in the table because it appears as significantly altered in more than one comparison (*m/zs* marked with an asterix). The identity of a sub-set of the metabolites was confirmed, using tandem mass spectrometry and matching the fragmentation pattern to a reference standard, are listed in Table 3.

m/z	Mass error (ppm)	FC	Comparison	p-value	Metabolite	Formula	Mode	Adduct
267.146*	1	2.080	Early (I and II)/Late (III)	2.88E-02	Leu His	C12H20N4O3	Negative	[M-H]-
303.233	1	0.140		6.24E-03	Arachidonic Acid	C20H32O2	Negative	[M-H]-
403.194*	1	4.152		7.90E-04	Glu Thr Arg	C15H28N6O7	Negative	[M-H]-
404.197	0	1.934		1.02E-03	Ala Gly Lys Met	C16H31N5O5S	Negative	[M-H]-
405.193	1	1.594		8.32E-04	Met Thr Arg	C15H30N6O5S1	Negative	[M-H]-
417.199	0	2.686		2.63E-04	Ala Leu Thr Asp	C17H30N4O8	Negative	[M-H]-
417.210*	0	1.118		3.13E-04	Ala Lys Asn Ser	C16H30N6O7	Negative	[M-H]-
418.208	3	1.748		3.59E-04	Trp Ser Lys	C20H29N5O5	Negative	[M-H]-
418.213*	0	9.460		4.17E-04	Ala Ala Lys Met	C17H33N5O5S	Negative	[M-H]-
431.179	0	3.203		7.69E-04	Ala Glu Val Asp	C17H28N4O9	Negative	[M-H]-
478.294	0	43.213		2.45E-02	PE(18:1/0:0)	C23H46NO7P	Negative	[M-H]-
480.309*	2	0.267		1.48E-02	PE(18:0/0:0)	C23H48NO7P	Negative	[M-H]-
485.282	4	3.028		2.28E-02	Ala Ile Gln Arg	C20H38N8O6	Negative	[M-H]-
524.278	0	0.106		3.33E-04	LysoPE(0:0/22:6)	C27H44NO7P	Negative	[M-H]-
526.293	1	0.390		2.44E-03	LysoPE(0:0/22:5)	C27H46NO7P	Negative	[M-H]-
566.346*	1	0.163		3.50E-02	PS(21:0/0:0)	C27H54NO9P	Negative	[M-H]-
606.074	0	0.377		2.98E-02	UDP-N-acetyl-D-galactosamine	C17H27N3O17P2	Negative	[M-H]-
665.314	1	1.636		9.71E-05	Lys Asp Tyr Glu Leu	C30H46N6O11	Negative	[M-H]-
746.513	0	2.387		2.78E-04	PE(20:5/P-18:1)	C43H74NO7P	Negative	[M-H]-
762.511*	3	2.035		8.36E-03	PE(16:0/22:6)	C43H74NO8P	Negative	[M-H]-
768.552	3	1.507		1.68E-02	PC(16:0/19:3)	C43H80NO8P	Negative	[M-H]-
772.528*	1	0.406		1.86E-03	PE(22:6/P-18:1)	C45H76NO7P	Negative	[M-H]-
774.542*	3	0.025		5.52E-03	PE(P-18:0/22:6)	C45H78NO7P	Negative	[M-H]-
779.563	4	0.144		5.74E-03	PA(20:0/22:4)	C45H81O8P	Negative	[M-H]-
788.526*	2	15.280		2.21E-02	PE(18:1/22:6)	C45H76NO8P	Negative	[M-H]-
803.563	4	11.292		3.35E-02	PA(22:0/22:6)	C47H81O8P	Negative	[M-H]-
824.543*	1	0.497		4.44E-02	PS(17:0/22:4)	C45H80NO10P	Negative	[M-H]-
828.575*	0	2.069		3.40E-04	PS(17:0/22:2)	C45H84NO10P	Negative	[M-H]-
851.562	4	0.127		4.82E-04	PI(13:0/22:0)	C44H85O13P	Negative	[M-H]-
852.574	2	1.570		5.41E-03	PS(19:0/22:4)	C47H84NO10P	Negative	[M-H]-
856.608	1	2.441		7.37E-04	PS(19:0/22:2)	C47H88NO10P	Negative	[M-H]-
858.660	0	3.101		1.13E-02	PS(P-20:0/22:0)	C48H94NO9P	Negative	[M-H]-
288.290	0	0.489		2.16E-04	C17 Sphinganine	C17H37NO2	positive	[M+H]+
415.213*	1	0.288		2.52E-05	Lys Met His	C17H30N6O4S1	positive	[M+H]+
454.179*	0	0.369		2.99E-05	Cys Cys Lys Thr	C16H31N5O6S2	positive	[M+H]+
665.419*	2	1.606		1.90E-03	PA(14:1/20:5)	C37H61O8P	positive	[M+H]+

705.592	1	3.371		1.84E-02	SM(d18:0/16:0)	C39H81N2O6P	positive	[M+H]+
780.554*	0	0.331		3.13E-03	PC(16:0/20:5)	C44H78NO8P	positive	[M+H]+
806.571*	1	3.081		7.86E-04	PC(16:0/22:6)	C46H80NO8P	positive	[M+H]+
806.571*	1	0.019		5.34E-03	PE(22:6/19:0)	C46H80NO8P	positive	[M+H]+
305.248	1	19,139	Early (I and II)/N	2.77E-03	5,8,11-eicosatrienoic acid	C20H34O2	Negative	[M-H]-
436.283	1	16.302		1.53E-05	PE(P-16:0/0:0)	C21H44NO6P	Negative	[M-H]-
464.314	1	10.907		3.10E-02	PC(P-15:0/0:0)	C23H48NO6P	Negative	[M-H]-
480.309*	0	15.617		1.25E-05	PE(18:0/0:0)	C23H48NO7P	Negative	[M-H]-
502.290*	3	8.916		2.05E-04	Thr Thr Gly Leu Ile	C22H41N5O8	Negative	[M-H]-
506.323	4	9		2.56E-03	PC(17:1/0:0)	C25H50NO7P	Negative	[M-H]-
538.347*	0	0.236		4.53E-02	Lys Lys Lys His	C24H45N9O5	Negative	[M-H]-
541.333	4	14.347		2.20E-02	Val Ile Pro Lys Ser	C25H46N6O7	Negative	[M-H]-
553.286*	1	36.245		3.20E-02	Asp His Lys Arg	C22H38N10O7	Negative	[M-H]-
555.272	2	9.7976		3.35E-03	PG(22:6/0:0)	C28H45O9P	Negative	[M-H]-
566.346	1	7.742		1.31E-03	PS(21:0/0:0)	C27H54NO9P	Negative	[M-H]-
599.320	0	62.527		3.01E-06	Lys Lys Tyr Tyr	C30H44N6O7	Negative	[M-H]-
608.245*	3	0.014		2.28E-20	His Gln Tyr Tyr	C29H35N7O8	Negative	[M-H]-
714.508*	0	10.866		6.47E-05	PE(16:0/18:2)	C39H74NO8P	Negative	[M-H]-
720.496	1	24.311		8.18E-04	PE(18:4/P-18:1)	C41H72NO7P	Negative	[M-H]-
740.521*	3	17.02		9.06E-06	PE(18:0/18:3)	C41H76NO8P	Negative	[M-H]-
742.539*	0	87.086		1.76E-08	PE(18:1/18:1)	C41H78NO8P	Negative	[M-H]-
747.517	1	2.337		2.16E-04	PG(16:0/18:1)	C40H77O10P	Negative	[M-H]-
748.528*	0	0.200		2.79E-06	PE(20:4/P-18:1)	C43H76NO7P	Negative	[M-H]-
750.544	0	0.454		8.50E-10	PE(O-16:0/22:5)	C43H78NO7P	Negative	[M-H]-
762.511*	3	2.658		3.11E-05	PE(16:0/22:6)	C43H74NO8P	Negative	[M-H]-
764.527*	4	12.513		1.08E-05	PC(15:0/20:5)	C43H76NO8P	Negative	[M-H]-
772.528*	1	0.358		1.56E-02	PE(22:6/P-18:1)	C45H76NO7P	Negative	[M-H]-
774.530*	1	73.131		2.79E-09	PS(13:0/22:1)	C41H78NO10P	Negative	[M-H]-
776.544*	1	169.37		2.46E-13	PS(13:0/22:0)	C41H80NO10P	Negative	[M-H]-
777.547*	3	10.311		1.64E-11	PA(20:1/22:4)	C45H79O8P	Negative	[M-H]-
788.526*	2	9.8885		2.28E-06	PE(18:1/22:6)	C45H76NO8P	Negative	[M-H]-
788.544*	0	0.263		5.69E-10	PS(18:0/18:1)	C42H80NO10P	Negative	[M-H]-
800.544*	1	6.613		2.29E-08	PS(15:0/22:2)	C43H80NO10P	Negative	[M-H]-
816.553*	2	0.008		4.16E-11	PE(20:1/22:6)	C47H80NO8P	Negative	[M-H]-
824.543*	1	8.711		1.14E-08	PS(17:0/22:4)	C45H80NO10P	Negative	[M-H]-
828.575*	0	2.794		5.41E-06	PS(17:0/22:2)	C45H84NO10P	Negative	[M-H]-
835.535*	0	21.918		2.27E-06	PI(16:0/18:1)	C43H81O13P	Negative	[M-H]-
839.577*	4	0.424		2.53E-05	PG(19:0/22:4)	C47H85O10P	Negative	[M-H]-
861.551*	0	90.4		2.90E-08	PI(18:1/18:1)	C45H83O13P	Negative	[M-H]-
863.565*	1	75.341		7.75E-07	PI(14:0/22:1)	C45H85O13P	Negative	[M-H]-
887.564*	1	43.357		1.15E-09	PI(16:0/22:3)	C47H85O13P	Negative	[M-H]-
256.264*	3	0.0136		5.36E-13	Palmitic amide	C16H33NO	Positive	[M+H]+
282.280*	4	0.019		2.55E-10	Oleamide	C18H35NO	Positive	[M+H]+
284.296*	3	0.088		1.01E-14	Stearamide	C18H37NO	Positive	[M+H]+
338.343*	2	0.035		5.99E-08	13Z-Docosenamide	C22H43NO	Positive	[M+H]+

369.353	3	28.704		1.16E-04	3-Deoxyvitamin D3	C27H44	Positive	[M+H]+
409.218*	2	0.112		5.43E-05	Gly His Pro Val	C18H28N6O5	Positive	[M+H]+
415.213*	1	130.06		1.37E-20	Lys Met His	C17H30N6O4S1	Positive	[M+H]+
451.166	3	0.370		1.64E-02	Trp Met Asp	C20H26N4O6S1	Positive	[M+H]+
454.179*	0	13.624		4.05E-18	Cys Cys Lys Thr	C16H31N5O6S2	Positive	[M+H]+
477.381*	4	209.13		3.10E-08	1-O-alpha-D-glucopyranosyl-1,2-eicosandiol	C26H52O7	Positive	[M+H]+
496.341	2	141.65		5.38E-03	PC(16:0/0:0)	C24H50NO7P	Positive	[M+H]+
522.356	1	75.614		1.46E-03	PC(O-16:1/2:0)	C26H52NO7P	Positive	[M+H]+
637.307*	3	0.028		1.65E-03	Glu Phe Arg Trp	C31H40N8O7	Positive	[M+H]+
675.544*	1	8.3		1.02E-02	SM(d18:1/14:0)	C37H75N2O6P	Positive	[M+H]+
705.582*	3	0.015		7.23E-04	PA(O-16:0/21:0)	C40H81O7P	Positive	[M+H]+
706.541*	4	454.26		1.61E-06	PE(16:0/17:0)	C38H76NO8P	Positive	[M+H]+
720.555*	1	31.796		5.81E-07	PE(17:0/17:0)	C39H78NO8P	Positive	[M+H]+
730.542*	4	286.63		7.60E-10	PC(14:0/18:2)	C40H76NO8P	Positive	[M+H]+
731.607*	1	0.014		1.44E-09	SM(d18:1/18:0)	C41H83N2O6P	Positive	[M+H]+
732.555*	1	5.55		1.28E-11	PC(14:0/18:1)	C40H78NO8P	Positive	[M+H]+
746.570*	1	111.54		1.23E-09	PE(18:0/18:1)	C41H80NO8P	Positive	[M+H]+
752.560*	1	0.064		1.40E-10	PE(20:3/P-18:1)	C43H78NO7P	Positive	[M+H]+
759.638*	1	0.116		1.22E-04	SM(d18:1/20:0)	C43H87N2O6P	Positive	[M+H]+
780.554*	0	82.53		2.45E-10	PC(16:0/20:5)	C44H78NO8P	Positive	[M+H]+
782.571	1	12.62		3.80E-02	PE(22:4/17:0)	C44H80NO8P	Positive	[M+H]+
787.670*	1	0.024		1.12E-08	SM(d18:1/22:0)	C45H91N2O6P	Positive	[M+H]+
806.571*	1	367.48		1.28E-05	PC(16:0/22:6)	C46H80NO8P	Positive	[M+H]+
811.666*	3	0.011		2.02E-09	SM(d18:2/24:1)	C47H91N2O6P	Positive	[M+H]+
813.686*	1	0.281		7.98E-10	SM(d18:1/24:1)	C47H93N2O6P	Positive	[M+H]+
814.634	2	21.334		3.64E-04	PC(16:0/22:2)	C46H88NO8P	Positive	[M+H]+
815.701	1	0.279		5.56E-05	SM(d18:1/24:0)	C47H95N2O6P	Positive	[M+H]+
832.586	1	19.434		8.70E-08	PC(18:1/22:6)	C48H82NO8P	Positive	[M+H]+
834.601	0	50.046		1.93E-03	PC(18:0/22:6)	C48H84NO8P	Positive	[M+H]+
836.611	1	10.669		7.67E-04	LacCer(d18:0/14:0)	C44H85NO13	Positive	[M+H]+
267.146*	1	43.913	**Late (III)/N**	4.77E-05	Histidylleucine	C12H20N4O3	Negative	[M-H]-
281.248	1	0.128		1.53E-05	2Z-octadecenoic acid	C18H34O2	Negative	[M-H]-
303.232	2	0.293		9.94E-04	8,11-eicosadiynoic acid	C20H32O2	Negative	[M-H]-
355.199	3	29.133		2.87E-02	Ala Ala Pro Val	C16H28N4O5	Negative	[M-H]-
397.209	0	37.246		2.27E-02	Pro Pro Ser Val	C18H30N4O6	Negative	[M-H]-
399.224	1	31.782		1.65E-02	Ala Ile Pro Thr	C18H32N4O6	Negative	[M-H]-
403.194*	1	10.872		2.16E-16	Gly Lys Asn Ser	C15H28N6O7	Negative	[M-H]-
417.210*	0	363.29		1.03E-20	Ala Lys Asn Ser	C16H30N6O7	Negative	[M-H]-
418.213*	0	44.8		3.71E-22	Ala Ala Lys Met	C17H33N5O5S	Negative	[M-H]-
426.259	2	40.28		3.44E-03	Arg Pro Arg	C17H33N9O4	Negative	[M-H]-
432.188	2	0.019		4.67E-05	Ala Ala Ser Trp	C20H27N5O6	Negative	[M-H]-
443.253	3	32.89		2.53E-03	Ala Glu Ile Ile	C20H36N4O7	Negative	[M-H]-
449.150	0	0.032		2.31E-02	Trp Met Asp	C20H26N4O6S1	Negative	[M-H]-
471.280	1	13.68		1.68E-02	Ala Ala Arg Arg	C18H36N10O5	Negative	[M-H]-
480.309*	0	7.56		4.84E-02	PE(18:0/0:0)	C23H48NO7P	Negative	[M-H]-

499.360	2	3.134		2.82E-03	Ile Ile Lys Lys	C24H48N6O5	Negative	[M-H]-
501.281	4	0.465		1.58E-02	Ala Glu Lys Arg	C20H38N8O7	Negative	[M-H]-
502.290*	3	21.16		1.51E-02	Thr Thr Gly Leu Ile	C22H41N5O8	Negative	[M-H]-
530.315	4	51.346		1.78E-07	Ile Ile Met Arg	C23H45N7O5S	Negative	[M-H]-
538.347*	0	0.236		9.44E-04	His Lys Lys Lys	C24H45N9O5	Negative	[M-H]-
553.286*	1	37.92		2.30E-06	Asp His Lys Arg	C22H38N10O7	Negative	[M-H]-
558.339	3	28.384		1.28E-04	Glu Lys Lys Arg	C23H45N9O7	Negative	[M-H]-
573.330	0	94.831		2.28E-04	Ile Met Arg Arg	C23H46N10O5S	Negative	[M-H]-
574.323	3	42.82		6.99E-05	Lys Thr Glu Lys Ala	C24H45N7O9	Negative	[M-H]-
599.319	0	38.788		9.18E-04	Lys Lys Tyr Tyr	C30H44N6O7	Negative	[M-H]-
601.389	2	56.529		1.76E-03	PA(12:0/17:2)	C32H59O8P	Negative	[M-H]-
608.245*	3	0.014		4.75E-34	His Gln Tyr Tyr	C29H35N7O8	Negative	[M-H]-
687.544	0	0.323		2.33E-04	SM(d16:1/17:0)	C38H77N2O6P	Negative	[M-H]-
714.508*	0	17.872		3.48E-03	PE(16:0/18:2)	C39H74NO8P	Negative	[M-H]-
716.523	0	21.399		3.52E-03	PE(18:1/16:0)	C39H76NO8P	Negative	[M-H]-
724.528	1	2.184		2.50E-07	PE(18:2/P-18:1)	C41H76NO7P	Negative	[M-H]-
736.528	1	2.436		8.68E-05	PC(18:4/P-16:0)	C42H76NO7P	Negative	[M-H]-
740.521*	3	17.11		8.94E-03	PE(18:0/18:3)	C41H76NO8P	Negative	[M-H]-
742.539*	0	86.553		1.12E-03	PE(18:1/18:1)	C41H78NO8P	Negative	[M-H]-
748.528*	0	0.233		2.13E-04	PE(20:4/P-18:1)	C43H76NO7P	Negative	[M-H]-
761.568	3	3.7379		1.47E-02	PG(O-16:0/20:1)	C42H83O9P	Negative	[M-H]-
762.511*	3	2.400		1.08E-02	PE(16:0/22:6)	C43H74NO8P	Negative	[M-H]-
764.527*	4	20.241		1.17E-03	PC(15:0/20:5)	C43H76NO8P	Negative	[M-H]-
768.553	2	4.6468		4.49E-02	PC(16:0/19:3)	C43H80NO8P	Negative	[M-H]-
772.528*	1	0.110		1.74E-03	PE(22:6/P-18:1)	C45H76NO7P	Negative	[M-H]-
774.530*	1	79.805		1.88E-06	PS(13:0/22:1)	C41H78NO10P	Negative	[M-H]-
774.542*	3	0.405		2.22E-03	PE(P-18:0/22:6)	C45H78NO7P	Negative	[M-H]-
775.531	3	9.4641		9.31E-08	PA(20:2/22:4)	C45H77O8P	Negative	[M-H]-
776.544*	1	153.81		9.32E-09	PS(13:0/22:0)	C41H80NO10P	Negative	[M-H]-
777.547*	3	15.165		4.10E-08	PA(20:1/22:4)	C45H79O8P	Negative	[M-H]-
788.526*	2	8.1507		1.30E-02	PE(18:1/22:6)	C45H76NO8P	Negative	[M-H]-
788.544*	0	0.494		7.83E-07	PS(18:0/18:1)	C42H80NO10P	Negative	[M-H]-
800.544*	1	3.9045		1.12E-03	PS(15:0/22:2)	C43H80NO10P	Negative	[M-H]-
816.553*	2	0.093		5.24E-09	PE(20:1/22:6)	C47H80NO8P	Negative	[M-H]-
824.543*	1	3.403		8.41E-04	PS(17:0/22:4)	C45H80NO10P	Negative	[M-H]-
835.535*	0	19.3		3.92E-04	PI(16:0/18:1)	C43H81O13P	Negative	[M-H]-
839.577*	4	0.424		3.19E-05	PG(19:0/22:4)	C47H85O10P	Negative	[M-H]-
860.607	4	8.6		4.22E-05	Galbeta1-4Glcbeta-Cer(d18:1/16:0)	C46H87NO13	Negative	[M-H]-
861.551*	0	56.74		1.68E-04	PI(18:1/18:1)	C45H83O13P	Negative	[M-H]-
863.565*	1	46.48		1.25E-05	PI(14:0/22:1)	C45H85O13P	Negative	[M-H]-
887.564*	1	69.912		5.25E-09	PI(16:0/22:3)	C47H85O13P	Negative	[M-H]-
256.264*	3	0.014		3.38E-10	Palmitic amide	C16H33NO	Positive	[M+H]+
282.280*	4	5.671E-04		3.11E-07	Oleamide	C18H35NO	Positive	[M+H]+
284.296*	3	0.088		4.15E-11	Stearamide	C18H37NO	Positive	[M+H]+
338.343*	2	4.402E-03		5.63E-16	13Z-Docosenamide	C22H43NO	Positive	[M+H]+

409.218*	2	0.112		2.55E-03	Gly His Pro Val	C18H28N6O5	Positive	[M+H]+
416.225	1	11.544		1.06E-06	Gly Pro Arg Ser	C16H29N7O6	Positive	[M+H]+
463.365	4	18.616		4.36E-03	1-O-alpha-D-glucopyranosyl-1,2-nonadecandiol	C25H50O7	Positive	[M+H]+
477.381*	4	53.203		1.70E-02	1-O-alpha-D-glucopyranosyl-1,2-eicosandiol	C26H52O7	Positive	[M+H]+
532.343	4	31.769		1.83E-03	Glu Lys Lys Lys	C23H45N7O7	Positive	[M+H]+
620.322	4	66.424		7.57E-04	Lys Thr Trp Trp	C32H41N7O6	Positive	[M+H]+
637.307*	3	0.028		1.25E-05	Glu Phe Arg Trp	C31H40N8O7	Positive	[M+H]+
665.419*	2	13.503		2.90E-03	PA(14:1/20:5)	C37H61O8P	Positive	[M+H]+
675.544*	1	5.551		3.37E-03	SM(d18:1/14:0)	C37H75N2O6P	Positive	[M+H]+
701.560	0	6.794		2.13E-03	SM(d16:1/18:1)	C39H77N2O6P	Positive	[M+H]+
705.582*	3	0.243		3.69E-04	PA(O-16:0/21:0)	C40H81O7P	Positive	[M+H]+
706.541*	4	314.64		5.80E-06	PE(16:0/17:0)	C38H76NO8P	Positive	[M+H]+
720.555*	1	4.2454		1.30E-04	PE(17:0/17:0)	C39H78NO8P	Positive	[M+H]+
730.542*	4	285.66		1.02E-06	PC(14:0/18:2)	C40H76NO8P	Positive	[M+H]+
731.607*	1	0.029		4.63E-08	SM(d18:1/18:0)	C41H83N2O6P	Positive	[M+H]+
732.555*	1	4.8033		8.81E-08	PC(14:0/18:1)	C40H78NO8P	Positive	[M+H]+
746.570*	1	89.507		4.05E-05	PE(18:0/18:1)	C41H80NO8P	Positive	[M+H]+
746.604	2	0.353		3.64E-02	PC(O-18:1/16:0)	C42H84NO7P	Positive	[M+H]+
752.560*	1	0.210		5.78E-06	PE(20:3/P-18:1)	C43H78NO7P	Positive	[M+H]+
752.561	2	0.070		2.03E-06	PE(O-16:0/22:5)	C43H78NO7P	Positive	[M+H]+
753.474	4	21.633		4.28E-06	PG(13:0/22:6)	C41H69O10P	Positive	[M+H]+
759.638*	1	0.116		2.35E-06	SM(d18:1/20:0)	C43H87N2O6P	Positive	[M+H]+
787.670*	1	9.620E-03		9.22E-07	SM(d18:1/22:0)	C45H91N2O6P	Positive	[M+H]+
794.606	0	0.408		2.35E-04	PC(O-18:1/20:4)	C46H84NO7P	Positive	[M+H]+
806.571*	1	2.334		3.59E-02	PC(16:0/22:6)	C46H80NO8P	Positive	[M+H]+
811.666*	3	0.291		1.14E-07	SM(d18:2/24:1)	C47H91N2O6P	Positive	[M+H]+
813.686*	1	0.465		4.38E-06	SM(d18:1/24:1)	C47H93N2O6P	Positive	[M+H]+
815.702	1	0.020		1.97E-04	SM(d18:1/24:0)	C47H95N2O6P	Positive	[M+H]+

Supplementary figure 1. Verification of stearamide using tandem MS. The identification of the metabolite stearamide (284.2946) in the samples was confirmed by performing MS/MS in the ESI postive mode with a collision energy of 40 V; the resultant fragmentation spectra was compared to that of a commercially available.

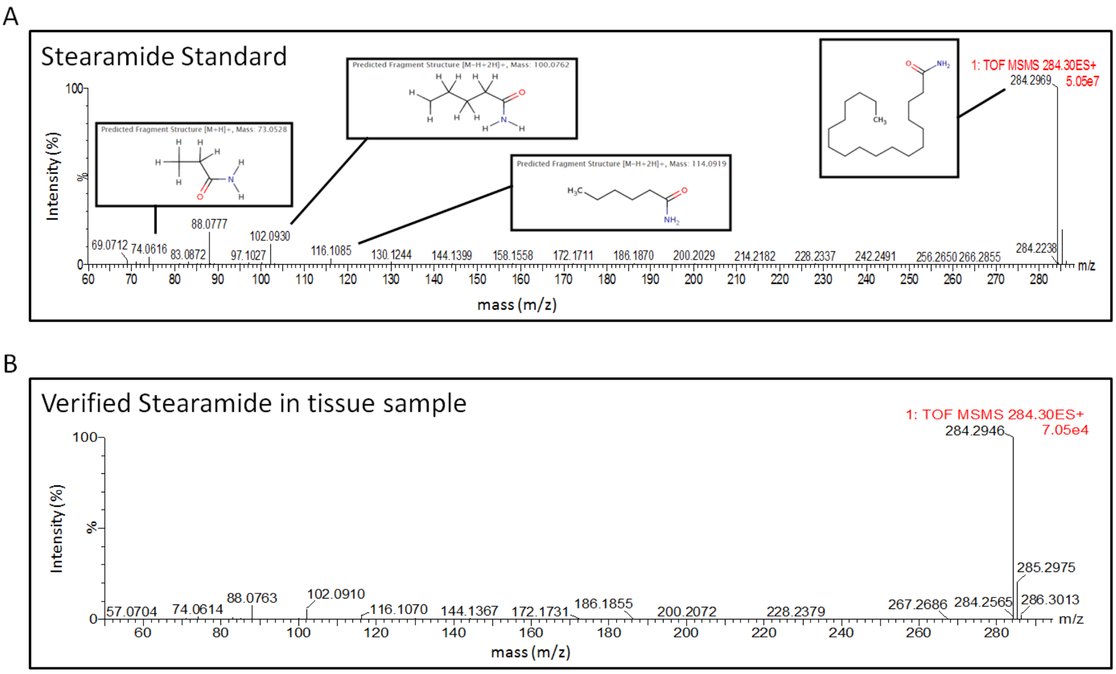

Supplementary figure 2. MS/MS structural verification of inosine. The identification of the metabolite inosine (267.0822) in the samples was confirmed by performing MS/MS in the ESI negative mode with a collision energy of 40 V; the resultant fragmentation spectra was compared to that available in Metlin web database.

A

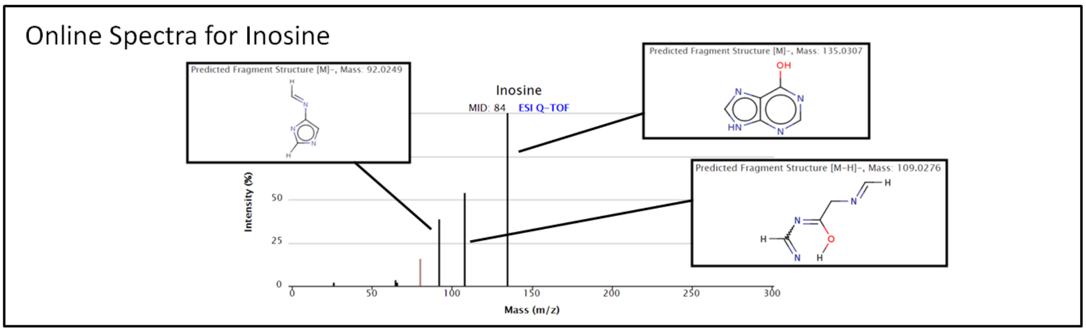

B

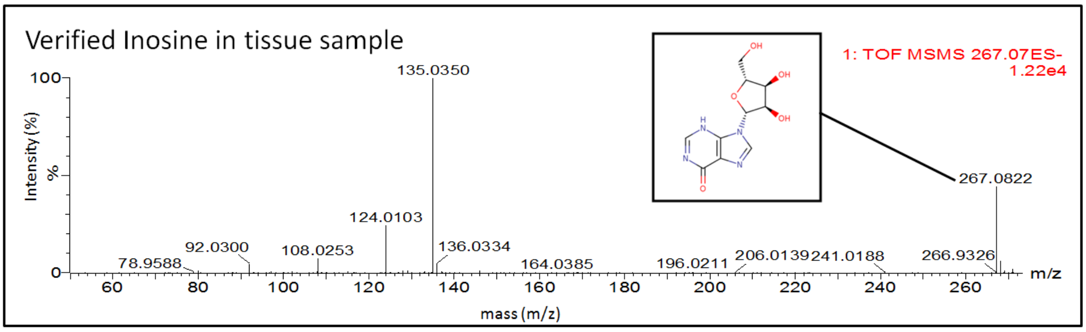

Supplementary figure 3. Metabolite panel as a predictor of EC progression. The realtive abundance of a panel of metabolites including arachidonic acid, PC (16:0/20:5), PE (22:6/P-18:1) and UDP-N-acetyl-D-galactosamine were significantly (p-v < 0.05) increased in late FIGO stages of EC compared to early stages while PC (16:0/22:6), PE (16:0/22:6) and PE (18:1/22:6) levels were significantly decreased.

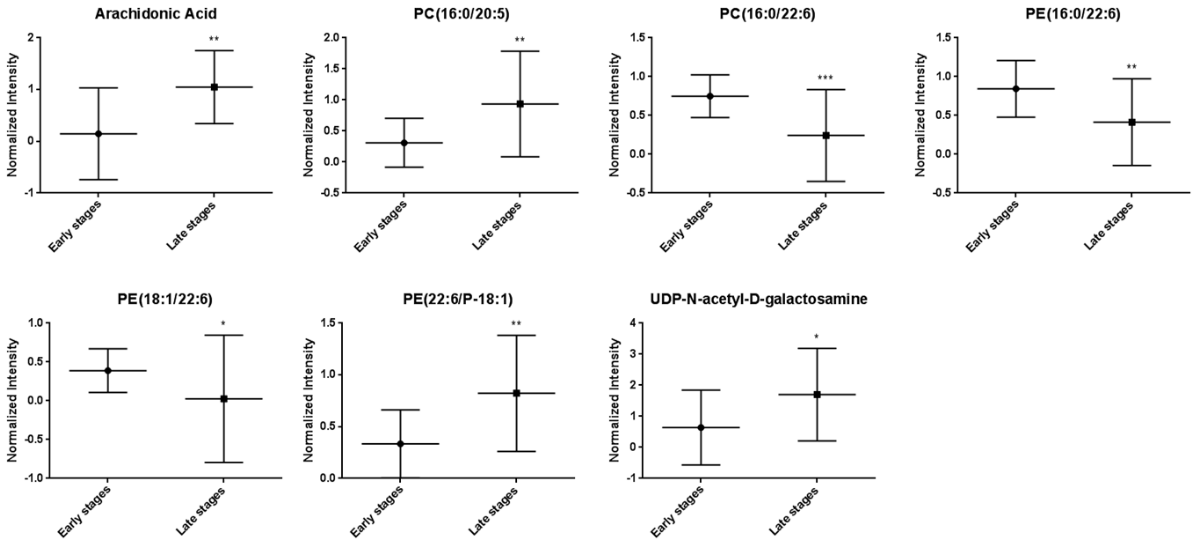

Supplementary figure 4. Staining profiles of ADAR proteins in EC tissues. Panel A. Representative section showing that ADAR1 expression is not detected in mitotic cells (black arrow). **Panel B.** ADAR1 specific staining of endothelial cells (black arrow). The same pattern was observed for ADAR2 antibody (data not shown). (100X).

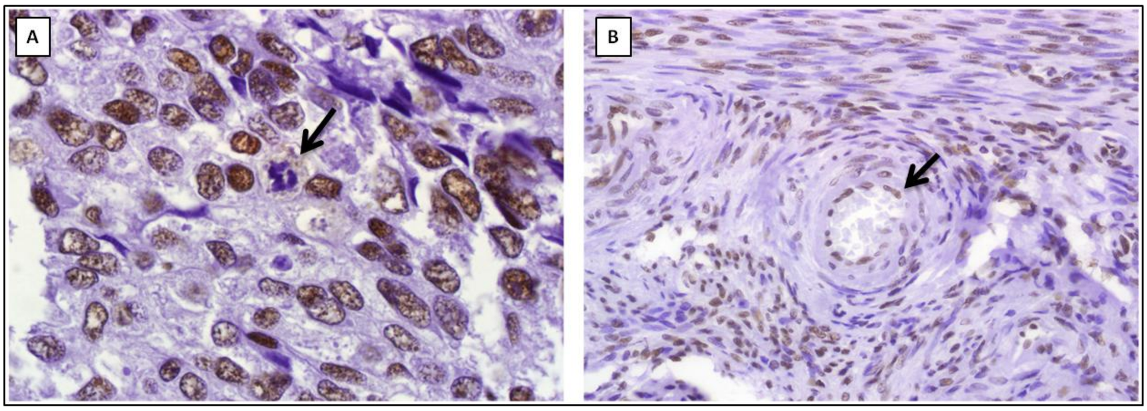

Supplementary figure 5. ROC curve analysis for IHQ data obtained for ADAR1 and ADAR2.

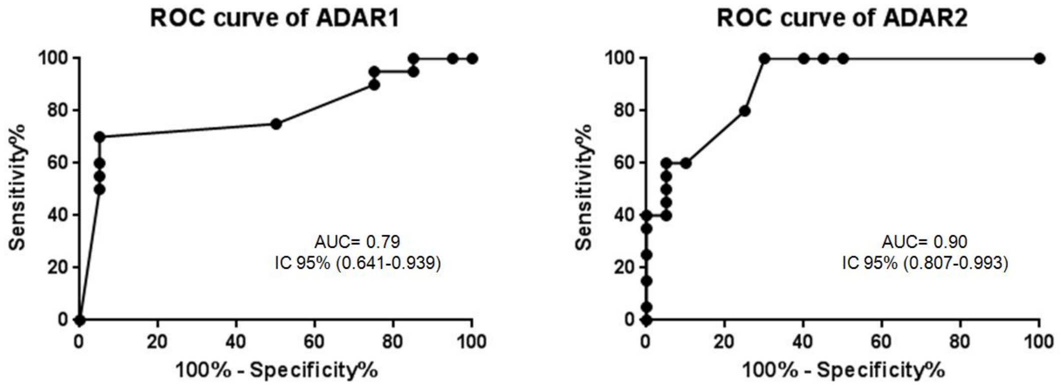

Supplementary figure 6. Representative graphic illustrating the transfection efficiency in three cell lines that was quantified as green fluorescence upon transfecting with siRNA-NC (**Panel A**) HEC-1A , (**Panel B**) Ishikawa and (**Panel C**) RL95-2 cells.

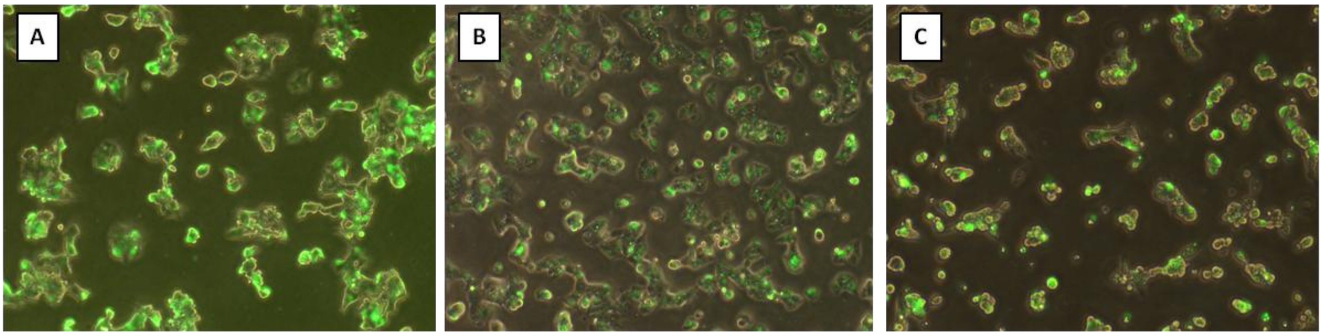

Supplementary figure 7. Panel A. Western Blot analysis showing the inhibition of ADARs protein expression. After 96 h of transfection of HEC-1A, Ishikawa and RL95-2 EC cell lines with siRNA-ADAR1, siRNA-ADAR2 or siRNA-NC, a decrease in protein abundance was observed in all cases. Naphtol blue staining of the membranes was used as a protein loading control. **Panel B. Immunofluorescence (IF) showing the decrease of ADARs protein expression**. IF was performed in the 3 cell lines after 96 h of transfection.

A

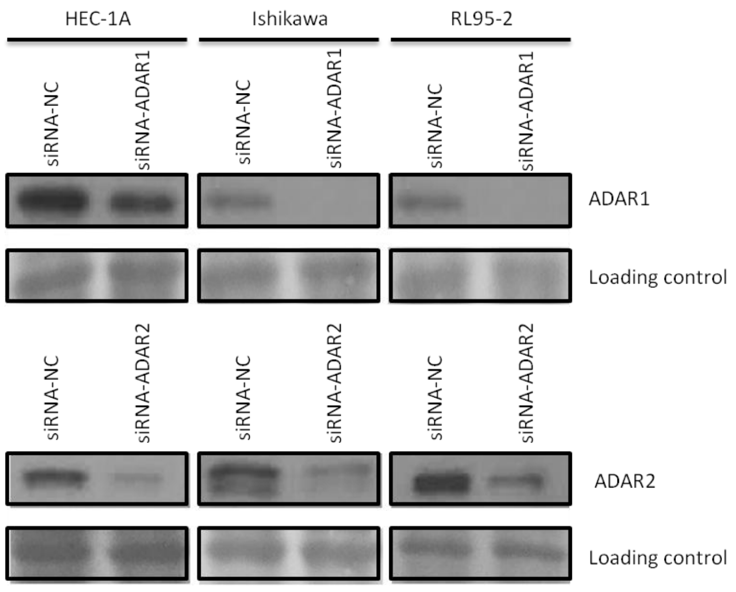

B

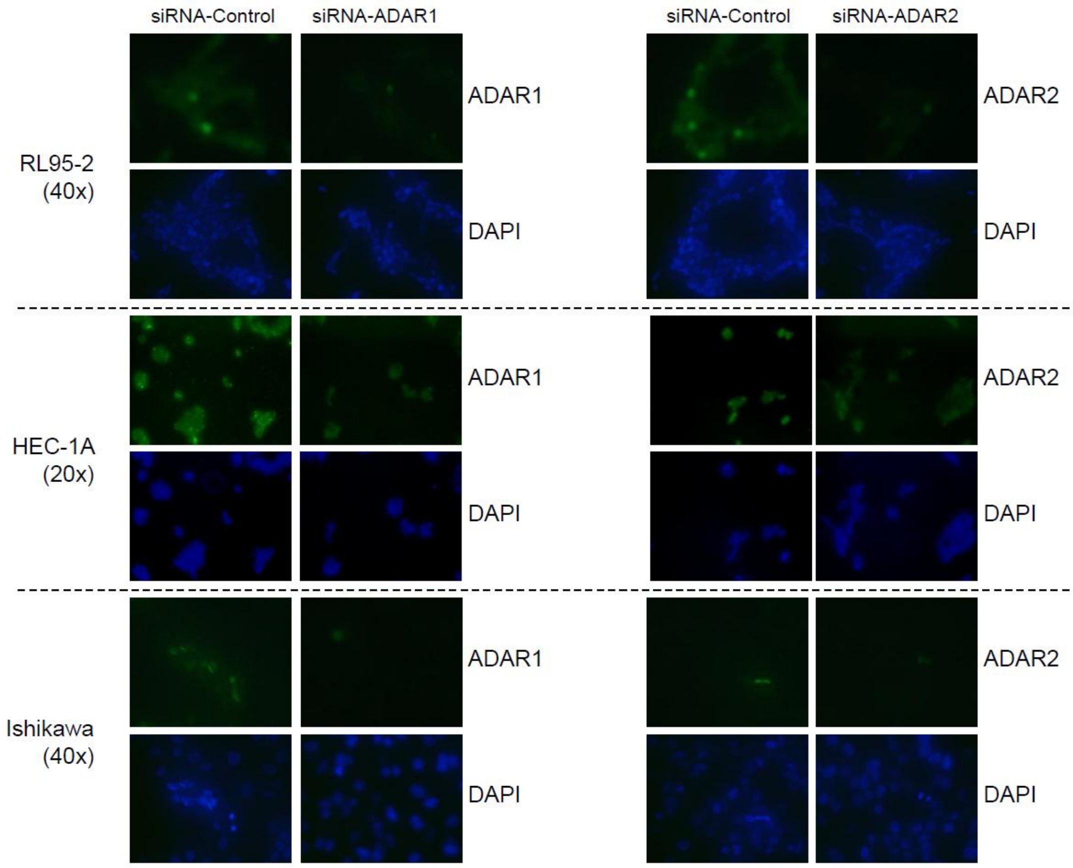

Supplementary figure 8. Panel A. Western Blot analysis showing the inhibition of ADAR2 protein expression. After 96 h of transfection of HEC-1A, Ishikawa and RL95-2 EC cell lines with siRNA-ADAR2_B, siRNA-ADAR2_C or siRNA-NC, a decrease in protein abundance was observed in all cases. Tubulin was used as a protein loading control. **Panel B. Functional assays revealing that ADAR2 presents oncogenic functions in vitro.** HEC-1A, Ishikawa and RL95-2 EC cell lines were used for the functional assays**.** Proliferation assay shows a significant decrease in cell viability (OD 590 nm) in the 3 cell lines when inhibiting ADAR2 expression with siRNA-ADAR2_B and siRNA-ADAR2_C. Apoptosis assay shows a significant increase in the apoptosis rate when silencing ADAR2 with both siRNAs. Wound healing assay indicating a significant decrease in the migration capabilities of the 3 EC cell lines (% of wound healing) when treating cells with siRNA-ADAR2_B and siRNA-ADAR2_C.

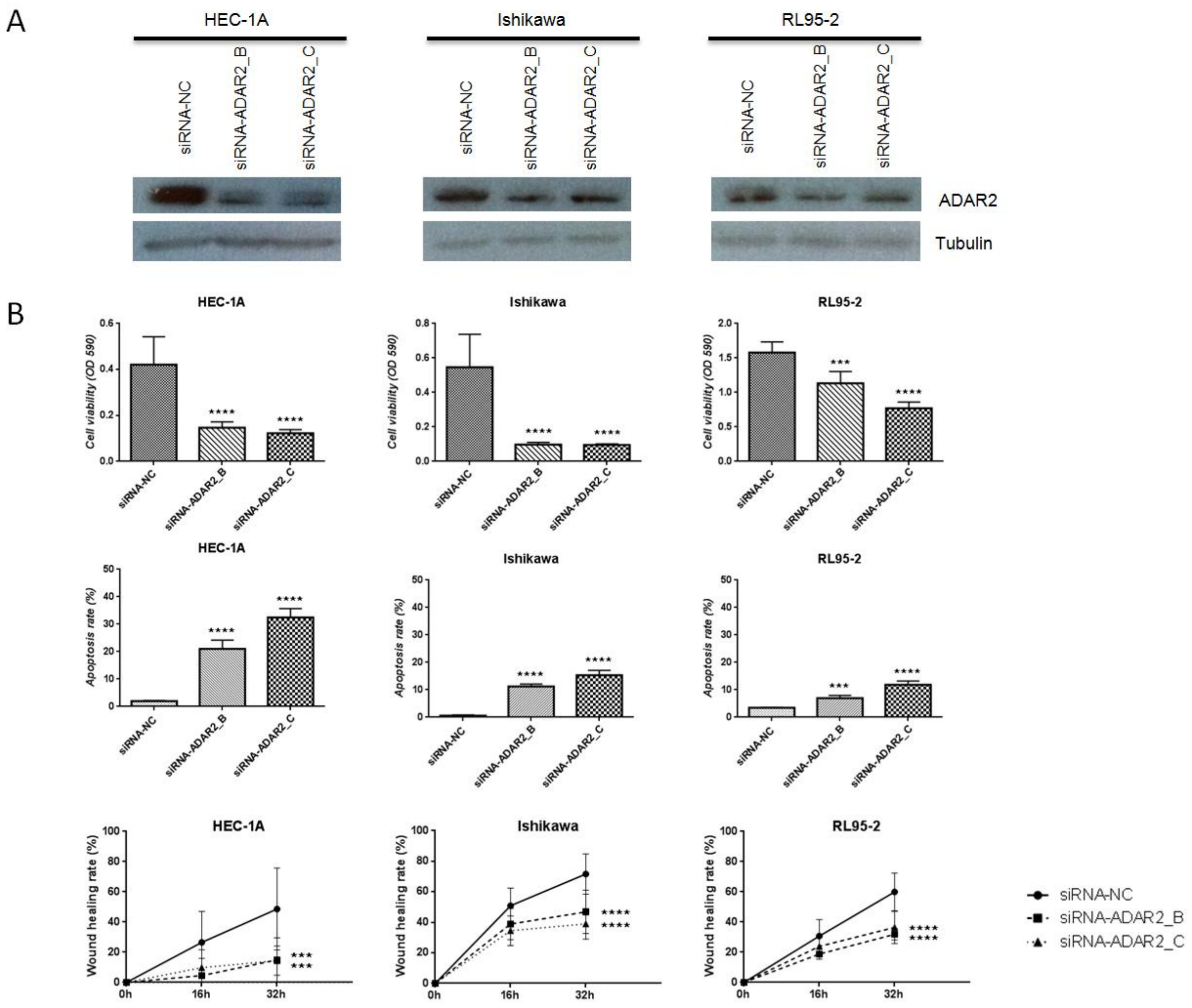

Supplement: Supplementary file 1 — Supplementary information [file 41598_2017_9169_MOESM1_ESM.pdf]
